# Transcriptomic and epigenomic analyses uncovered Lrrc15 as a contributing factor to cartilage damage in osteoarthritis

**DOI:** 10.1038/s41598-021-00269-8

**Published:** 2021-10-26

**Authors:** Purva Singh, Mengying Wang, Piali Mukherjee, Samantha G. Lessard, Tania Pannellini, Camila B. Carballo, Scott A. Rodeo, Mary B. Goldring, Miguel Otero

**Affiliations:** 1 Hospital for Special Surgery, HSS Research Institute, New York, NY 10021 USA; 2 School of Public Health, Xi’an Jiaotong University Health Science Center, Xi’an, China; 3 Weill Cornell Medicine, New York, NY 10021 USA; 4 Hospital for Special Surgery, Orthopedic Soft Tissue Research Program, HSS Research Institute, Room 603, 535 East 70th Street, New York, NY 10021 USA

**Keywords:** Genome-wide analysis of gene expression, Methylation analysis, Osteoarthritis, Cartilage, Musculoskeletal models, Animal disease models

## Abstract

In osteoarthritis (OA), articular chondrocytes display phenotypic and functional changes associated with epigenomic alterations. These changes contribute to the disease progression, which is characterized by dysregulated reparative processes and abnormal extracellular matrix remodeling leading to cartilage degradation. Recent studies using a murine model of posttraumatic OA highlighted the contribution of changes in DNA hydroxymethylation (5hmC) to OA progression. Here, we integrated transcriptomic and epigenomic analyses in cartilage after induction of OA to show that the structural progression of OA is accompanied by early transcriptomic and pronounced DNA methylation (5mC) changes in chondrocytes. These changes accumulate over time and are associated with recapitulation of developmental processes, including cartilage development, chondrocyte hypertrophy, and ossification. Our integrative analyses also uncovered that Lrrc15 is differentially methylated and expressed in OA cartilage, and that it may contribute to the functional and phenotypic alterations of chondrocytes, likely coordinating stress responses and dysregulated extracellular matrix remodeling.

## Introduction

Cartilage degradation is a hallmark of osteoarthritis (OA), but the mechanisms initiating cartilage destruction are still not clearly identified and no successful therapeutic intervention exists^1^. This knowledge gap is in part caused by the difficulty of identifying early-stage disease and of retrieving mechanistic information from early-stage human clinical material. Consequently, the design of adequate models that mimic aspects of the human pathology stands essential for the development of successful therapeutic approaches.

Articular chondrocytes are the unique cell type in articular cartilage and are responsible for maintaining its structural and functional integrity. During OA, chondrocytes undergo abnormal activation and phenotypic modulation, displaying features that resemble hypertrophy- and fibroblast-like phenotypes characterized by dysregulated expression and activities of matrix-degrading enzymes and abnormal production of structural matrix proteins^2^. Recent studies have reported changes in DNA methylation in human OA cartilage^3–6^, work in vivo and in vitro has highlighted the importance of a tight regulation of DNA methylation patterns for cartilage homeostasis and OA^7,8^, and a link between inflammation and epigenomic alterations in OA has been established^9^. These studies represent strong evidence that functional pathological alterations in methylation status do occur, are subsequently maintained, and have important roles in the phenotypic shift and functional alterations of OA chondrocytes.

DNA methylation on the fifth position of cytosine (5-methylcytosine, 5mC) is a stable epigenetic mark with important roles in development and maintenance of stable cellular phenotypes^10^. DNA methylation can be found at non-CpG sites but it is primarily restricted to CpG dinucleotides (cytosine-guanine repeats) in mammals^11^, and the DNA methyl transferases (DNMTs) preserve normal DNA methylation patterns^12,13^. Loss of methylation or abnormal methylation patterns contribute to OA^7^. Methylation can be lost passively or by an active process that involves the conversion of 5mC into 5-hydroxymethylcytosine (5hmC), which is also a stand-alone epigenetic mark with impact on OA disease^8,14^. Because DNA methylation is one of the principal mechanisms by which cells maintain dominant phenotypes, changes in DNA methylation patterns can lead to early phenotypic and functional changes in chondrocytes that contribute to OA onset and progression, as indicated by recent studies^7,8^.

To further dissect changes in 5mC and 5hmC during OA progression, we performed integrated RNA sequencing (RNAseq) and Reduced Representation Oxidative Bisulfite Sequencing (RRoxBS) analyses of microdissected murine cartilage following the destabilization of the medial meniscus (DMM), which mimics post-traumatic OA in humans^15,16^ and has also been used to assess the contribution of epigenetic changes to OA^8^.

## Results

### The progression of osteoarthritis after DMM is accompanied by transcriptional changes in articular cartilage

To evaluate genomics changes during the progression of OA we undertook an integrative approach whereby we analyzed (a) cartilage structural damage using histological approaches, (b) changes in gene expression over time using RNAseq, and (c) progressive alterations in 5mC and 5hmC DNA methylation patterns by RRoxBS.

**Figure 1:**
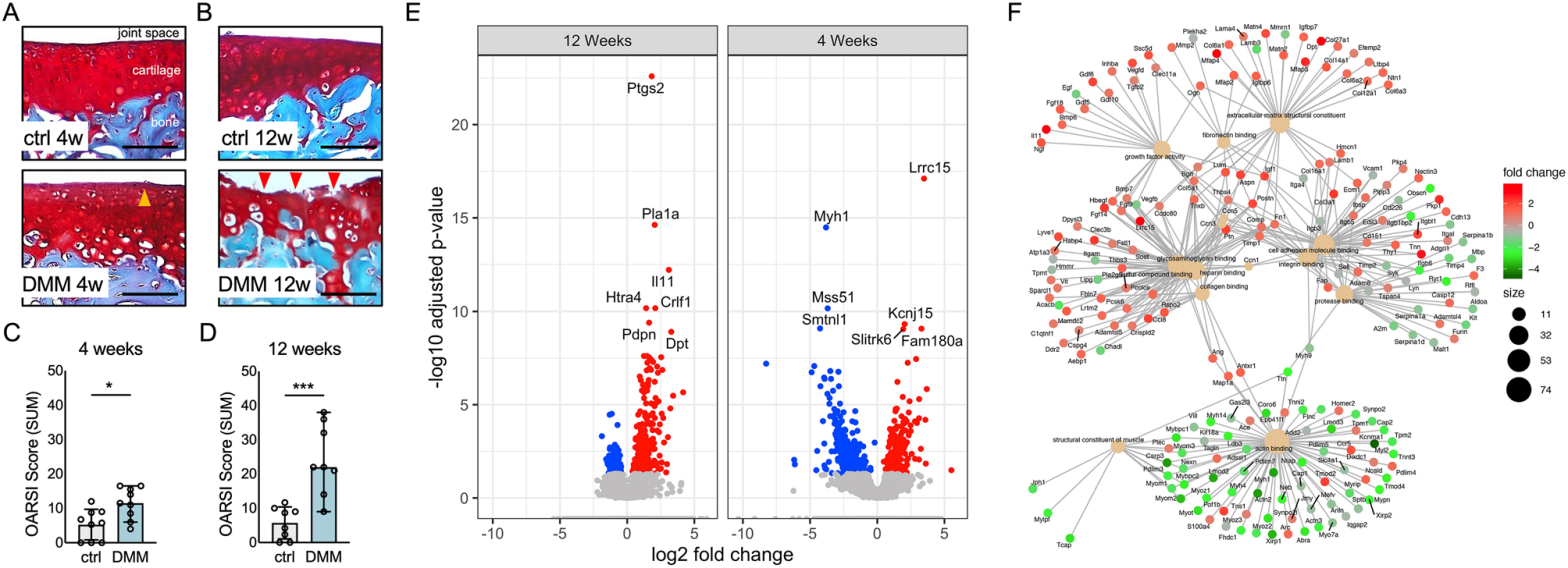
The progression of osteoarthritis after DMM is accompanied by transcriptional changes in articular cartilage. Representative Safranin O-stained histological sections of mouse cartilage at (**A**) 4 and (**B**) 12 weeks after surgical induction of OA (original magnification × 20, scale bar = 50 μm). Arrowheads indicate areas of proteoglycan depletion and minor fibrillation (orange, 4 weeks), or loss of surface and structural damage (red, 12 weeks). Graphs represent the OARSI (SUM) cartilage degradation scores for (**C**) 4 (n = 9/ea) and (**D**) 12 (n = 8/ea) weeks. *p < 0.05 and ***p < 0.001 by Mann–Whitney. (**E**) Volcano plot representing significantly differentially expressed genes (adjusted p-value < 0.05) identified by RNA-seq analyses of microdissected cartilage tissues retrieved at 4 and 12 weeks after DMM surgery (n = 3 per condition and per time point). Log fold-changes in the OA (DMM operated) vs. control limbs are shown for each time point. Red indicates high expression in the OA samples. Blue indicates high expression in the control samples. (**F**) A gene concept network of functional enrichment showing genes with increased (red) and decreased (green) expression in OA cartilage from top enriched functions in cartilage tissues after surgical induction of OA.

We performed DMM surgeries and analyzed tissues at 4 and 12 weeks after DMM to evaluate genomics changes in samples with clearly distinct cartilage structural damage, representing early (4 weeks) and established (12 weeks) disease. We evaluated tissues histologically to confirm the progression of OA after DMM. Figure 1A depicts the initial loss of proteoglycan staining and minor surface damage at 4 weeks, followed by the more evident fibrillation and structural changes in cartilage collected at 12 weeks after surgery (Fig. 1B). These changes were confirmed by histological OARSI SUM scores (Fig. 1C,D).

Next, we used RNAseq to evaluate changes in gene expression in total RNA extracts from microdissected cartilage collected at 4 and 12 weeks after DMM. Comparing DMM-operated (n = 3 per time-point) and non-operated control limbs (n = 3 per time-point) from the same mice, we identified 529 and 583 differentially expressed genes (DEGs, Benjamini-Hochberg (BH) adjusted p-value < 0.05) at 4 and 12 weeks after DMM, respectively (Fig. 1E and Supplementary Table S1). We identified 474 DEGs unique to early OA (4 weeks), 528 DEGs unique to more established OA (12 weeks), and 55 DEGs common to both 4 and 12 weeks (Supplementary Figure S1). In addition to uncovering novel genes with potential relevance to the early phases of OA disease (including *Lrrc15* or *Lrrc1*7), our RNAseq analyses confirmed changes in the expression of genes with known associations to OA, including *Aspn*, *Adamts16, Mmp3* and *Ptgs2* (see Supplementary Table S1 and references^8,17–20^). Gene ontology (GO) analyses integrating DEGs at 4 and 12 weeks showed enrichment of biological processes, cell components, and molecular functions relevant to cartilage development, ossification, and hypertrophy in OA (see Supplementary Figure S2 and Supplementary Table S2), consistent with previous reports^7–9^. Category network (cnet) analyses confirmed these observations and highlighted the contribution to OA of networks relevant to ECM assembly and signaling (Fig. 1F).

### The progression of osteoarthritis after DMM surgery is accompanied by changes in methylation patterns in articular cartilage

OA chondrocytes undergo phenotypic and functional alterations that are in part driven by changes in DNA methylation^2^, including changes in 5hmC following DMM^8^. To evaluate how the structural and transcriptomic changes following DMM are associated with changes in 5mC and 5hmC, we conducted Reduced Representation Oxidative Bisulfite Sequencing (RRoxBS) analyses^21,22^. These analyses uncovered significant differences in hyper- and hypo-methylation at 4 and 12 weeks after DMM (Supplementary Figure S3). Using at least a 25% methylation difference and q-value of less than 0.05 in comparisons between DMM and control samples, we identified 842 differentially methylated 5mCs and 318 5hmCs at 4 weeks after DMM, and a pronounced increase in the number of differentially methylated cytosines (DMCs) at 12 weeks. This was particularly evident for 5mCs, with 3614 differentially methylated 5mCs at 12 weeks and 480 differentially methylated 5hmCs (Supplementary Table S3).

**Figure 2:**
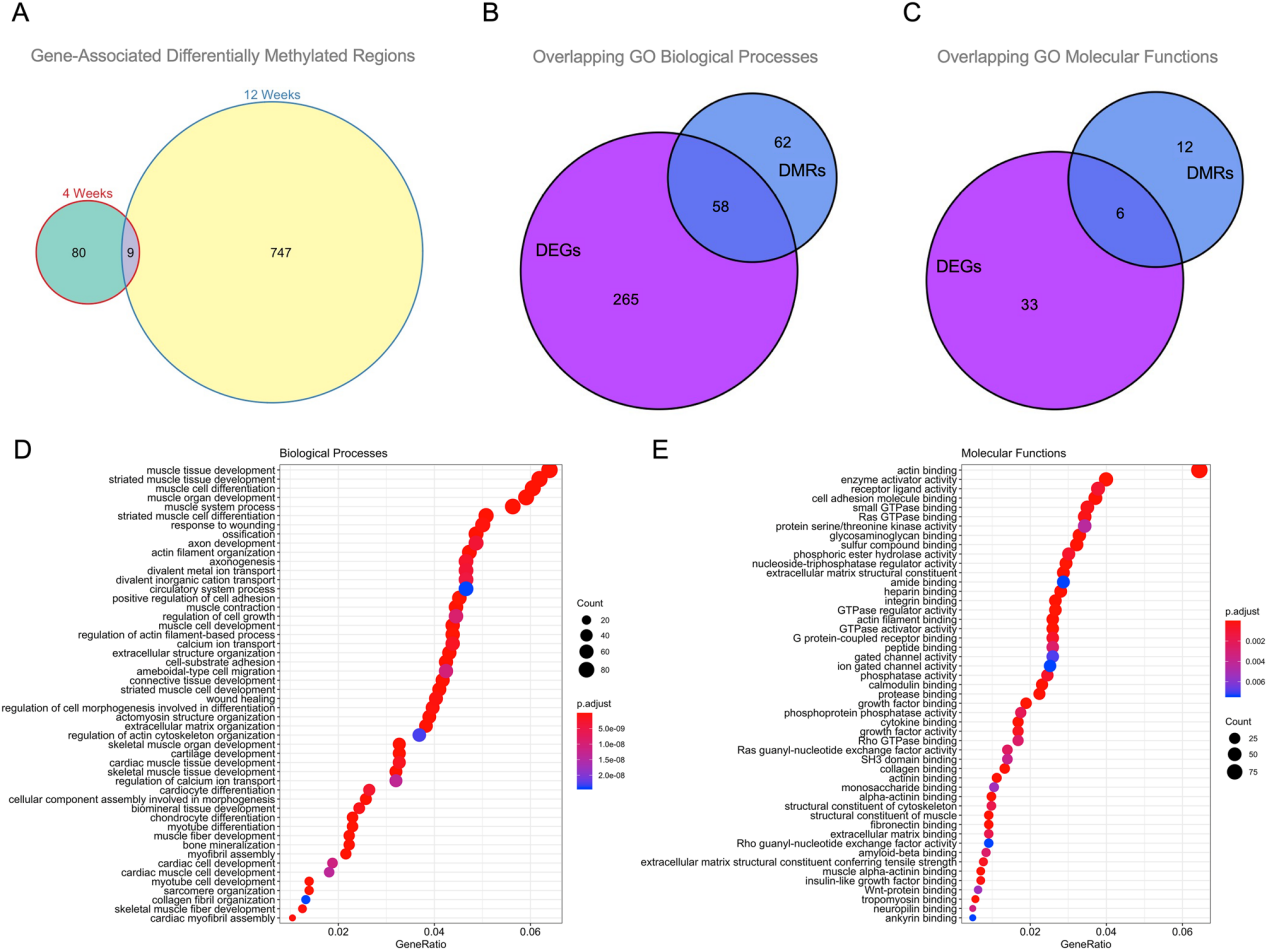
RRoxBS analyses identified changes in 5mC and 5hmC in mouse cartilage isolated after surgical induction of OA. (**A**) Changes in gene-associated, differentially methylated regions (DMRs, 25% difference in methylation and q value < 0.05) in microdissected cartilage at 4 and 12 weeks after induction of OA. Overlapping significantly enriched (**B**) Biological Processes and (**C**) Molecular Functions comparing gene expression (RNA-seq) and DNA methylation (RRoxBS, 5mC). Representation of the (**D**) Biological Processes (top 50) and (**E**) Molecular Functions significantly enriched (FDR < 0.05) for all significant genes, using differentially methylated regions (DMRs) and differentially expressed genes (DEGs) in OA vs. non-OA mouse cartilage samples.

The impact of 5hmC in OA has been reported using the DMM model and *Tet1* deficient mice^8^. Thus, we focused on true methyl data (5mC) for our downstream analyses. First, we used 5mC data to identify differentially methylated regions (DMRs). We defined DMR as a genomic region with at least 3 CpGs within 100 bp, where at least 1 CpG is significantly differentially methylated (25% 5mC methylation difference and q value < 0.01). The overall differential methylation of 5mC averaged at least 20% across all the CpGs in this DMR. We identified 89 DMRs associated with 90 unique gene symbols at 4 weeks, and 756 DMRs associated with 489 unique gene symbols at 12 weeks, with 9 common DMRs (Fig. 2A and Supplementary Table S4). Functional integration of DEGs and DMRs at 4 and 12 weeks via GO categories revealed 58 overlapping biological processes (Fig. 2B), and 6 overlapping molecular functions (Fig. 2C) significantly enriched in OA cartilage, including collagen binding (with *Aspn*, *Itgb1*, *Lrrc15*, or *Smad3*), extracellular matrix structural constituent (including *Aspn*, *Col4a1*, *Col5a1*, or *Col6a3*), or integrin binding (with *Itgb1*, *Itgb3*, *Itgb4* or *Timp2*). See Table 1 for a list of selected GO categories, and Supplementary Table S5 for a complete list of GO categories. Functional analyses using the 4- and 12-week significant genes based on DMR and DEG data showed that biological processes (Fig. 2D) and molecular functions (Fig. 2E) relevant to ECM constituents, enzymatic binding and activity, or growth factor and cytokine binding were differentially enriched in the OA cartilage samples.

**Table 1:** Functional integration of differentially expressed genes and differentially methylated regions.

ID	Description	p.adjust.DMRs	p.adjust.DEGs
**Biological processes**			
GO:0030198	Extracellular matrix organization	4.94528E−06	1.36E−09
GO:0043062	Extracellular structure organization	4.94528E−06	2.90E−10
GO:0031032	Actomyosin structure organization	2.83234E−05	1.20E−13
GO:0010769	Regulation of cell morphogenesis involved in differentiation	4.11917E−05	4.38219E−05
GO:0031589	Cell-substrate adhesion	4.97571E−05	1.76E−08
GO:0032970	Regulation of actin filament-based process	5.90698E−05	3.33326E−06
**Molecular functions**			
GO:0005518	Collagen binding	0.001358102	1.06334E−05
GO:0003779	Actin binding	0.001358102	2.89E−19
GO:0008201	Heparin binding	0.001708286	4.36E−11
GO:0005201	Extracellular matrix structural constituent	0.002977544	5.50E−15
GO:0005178	Integrin binding	0.003146782	1.53E−12
GO:0050839	Cell adhesion molecule binding	0.005629405	1.85E−12

Summary of the Gene Ontology (GO) Biological Processes (BP) and Molecular Functions (MF) categories identified using differentially expressed genes (DEGs) and differentially methylated regions (5mC, DMRs). The table includes 6 of the top Biological Processes (out of a total of 58) and all identified Molecular Functions, including the adjusted p values (p.adjust) for DMRs and DEGs.

Along with our transcriptomic data (Fig. 1), these epigenomic changes are in agreement with reports that highlight the importance to OA of biological processes and molecular functions relevant to ECM assembly and signaling^7^. Together, our transcriptomic and epigenomic analyses confirmed the changes in gene expression and DNA methylation reported using human samples and murine tissues^6,7,19,23–27^ and further confirm that the progression of OA is accompanied by time-dependent changes in the transcriptome and DNA methylome of articular cartilage, in agreement with recent reports^8^.

### Integrative analyses and interrogation against human datasets uncovered the *Lrrc15* gene as a potential novel target in early OA

Structural changes in established OA can affect downstream genomics analyses due to the loss of specific chondrocyte (sub)populations^28^. Thus, we focused on the 4-week samples for additional comparisons, aiming to minimize the impact of cartilage loss and to identify early changes.

**Figure 3:**
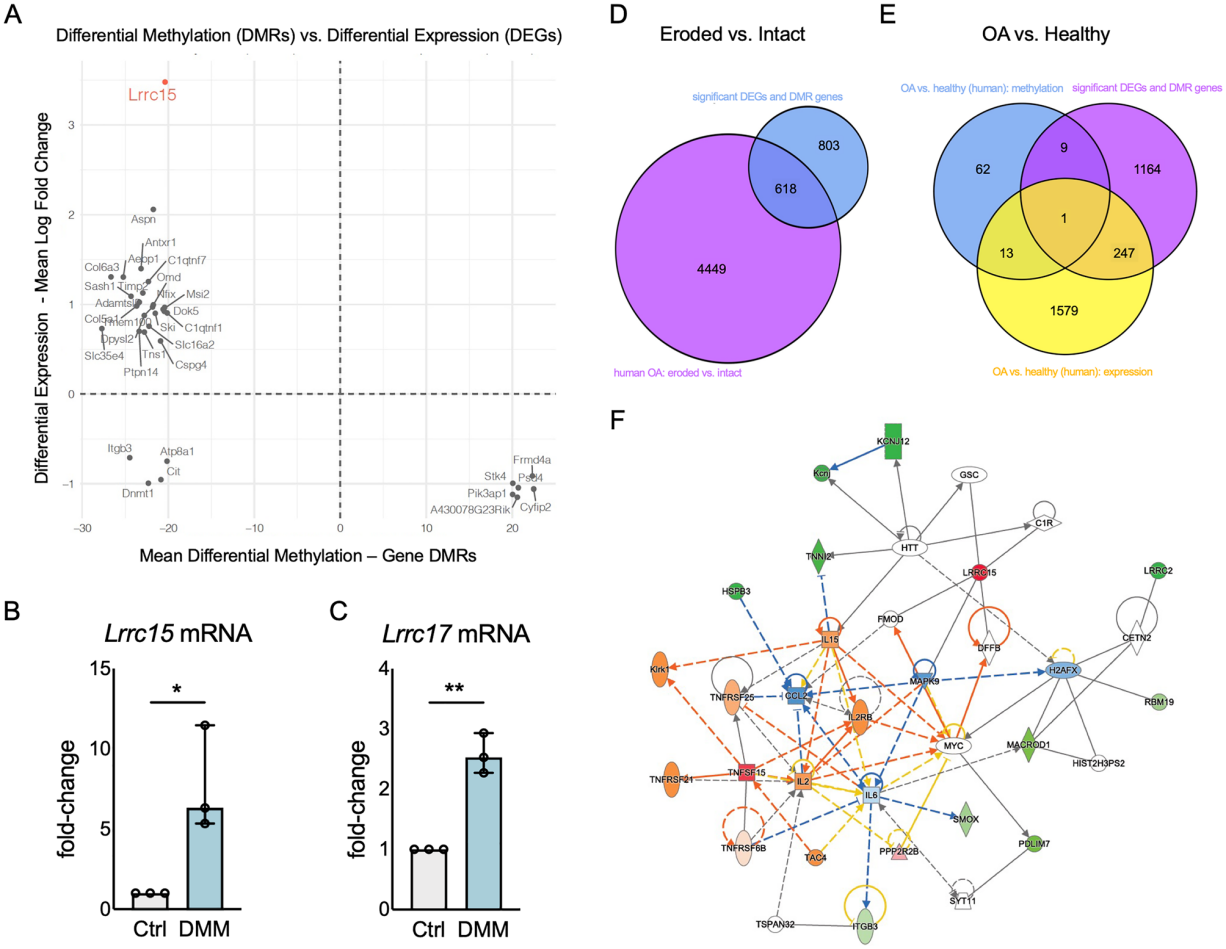
Lrrc15 is differentially methylated and differentially expressed in mouse OA cartilage. (**A**) Co-representation of differential expression (y axis, shown as mean Log Fold Change) and differential methylation (x axis, shown as mean differential methylation in gene associated DMRs) of genes with significant differential expression and methylation at 4 and 12 weeks. Lrrc15 is highlighted in red as the gene with the highest correlation between increased expression and reduced 5mC at 4 weeks. RTqPCR analyses of (**B**) Lrrc15 and (**C**) Lrrc17 mRNA in mouse cartilage samples at 4 weeks after surgical induction of OA (n = 3/ea). Data are shown as fold-change vs. controls (set as 1). *p < 0.05 by t-test. Venn diagram depicting unique and overlapping differentially expressed genes (DEGs) and differentially methylated regions (DMRs) obtained from our dataset using microdissected cartilage after DMM and published human datasets from human OA cartilage using (**D**) structurally intact and eroded cartilage and (**E**) healthy and OA cartilage samples. (**F**) Network analyses representing the interaction of Lrrc15 with other genes with differential methylation and expression at 4 weeks after surgical induction of OA. Circles indicate complexes or groups; squares indicate cytokines; diamonds represent enzymes; vertical ovals indicate transmembrane receptors; horizontal ovals indicate transcription regulators. A solid line connecting molecules indicates a direct interaction, and dotted lines indicate indirect interactions. Genes coloured in red are significantly overexpressed and in green are significantly underexpressed. A sub-pathway for upstream and downstream activity prediction is also highlighted, where orange is predicted activation and blue is predicted inhibition. A detailed legend for molecule shapes and interaction is available at https://qiagen.secure.force.com/KnowledgeBase/articles/Basic_Technical_Q_A/Legend.

First, we performed correlative analyses using our RNAseq and RRoxBS data to identify genes with changes in expression and DNA (5mC) methylation at 4 and 12 weeks after DMM (Fig. 3A). Leucine Rich Repeat Containing (Lrrc) 15 emerged as the gene with the strongest inversal correlation between hypomethylation and increased gene expression at 4 weeks in our dataset (Fig. 3A, Lrrc15 highlighted in red). We confirmed increased *Lrrc15* expression at 4 weeks after DMM by RTqPCR (Fig. 3B), which also confirmed changes in *Lrrc17* mRNA (Fig. 3C), another LRRC gene identified by RNAseq as differentially expressed in human and mouse OA tissues (Supplementary Figure S1), but having no change in 5mC methylation.

To identify clinically relevant changes, we interrogated our data against available human datasets. These comparisons highlighted notable disease stage- and platform-dependent differences within human datasets (Supplementary Figure S4). Comparisons with HuGENet (Supplementary Figure S4) identified 28 overlapping genes (out of 168 OA-associated genes), including 9 genes with gene associated-DMRs (Havcr2, Ncor2, Aspn, Tnfrsf11b, Smad3, Tcf7l1, Lrp5, Fos, and Pepd). We further separated the published datasets into two comparator groups: eroded vs. non-eroded OA cartilage^6,18,25,26,29,30^, with 618 overlapping genes (Fig. 3D), and healthy vs. OA cartilage^17,31–33^, with 248 DEGs (including LRRC15) and 10 DMR associated genes overlapping, and Runx1 as the gene at the intersect between methylation and expression in published human datasets and our mouse data (Fig. 3E). See Supplementary Table S6 for details.

These analyses confirmed that LRCC15 expression is increased in human OA cartilage samples, and suggest that differential expression of LRRC15 in OA cartilage may be partly driven by changes in DNA methylation. Our initial network analyses showed that *Lrrc15* is part of the collagen binding network enriched in OA (Fig. 1F). We mined our datasets to evaluate additional interactions of *Lrrc15* with differentially expressed or methylated genes at 4 weeks after DMM. The regulatory network of molecular interactions using ingenuity pathway shown in Fig. 3F represents the integration and interaction of *Lrrc15* in a molecular network including binding to extracellular matrix constituents (FMOD), transcriptional regulators (GSC, MAPK9) and nucleases (DFFB)^34^; and regulating pro-inflammatory cytokine pathways (increased IL6 and TNFSF15 expression and predicted regulation of IL2, IL2RB and IL15 activation via TNF and TNFR super family members), thus potentially contributing to remodeling, reparative, and developmental processes, as well as stress/inflammatory signaling and apoptosis^35–38^. All the five direct interactions for *Lrrc15* were protein–protein interactions^34^. Thus, our integrative analyses confirmed that the increased expression of *Lrrc15* is conserved in human and mouse OA cartilage and suggest a potential functional involvement of *Lrrc15* in OA disease, as part of a functional network that involves chondrocyte phenotypic changes and coordination of stress responses, including hypertrophic-like changes and extracellular matrix degradative processes.

### LRRC15 immunostaining in human and murine OA cartilage

**Figure 4:**
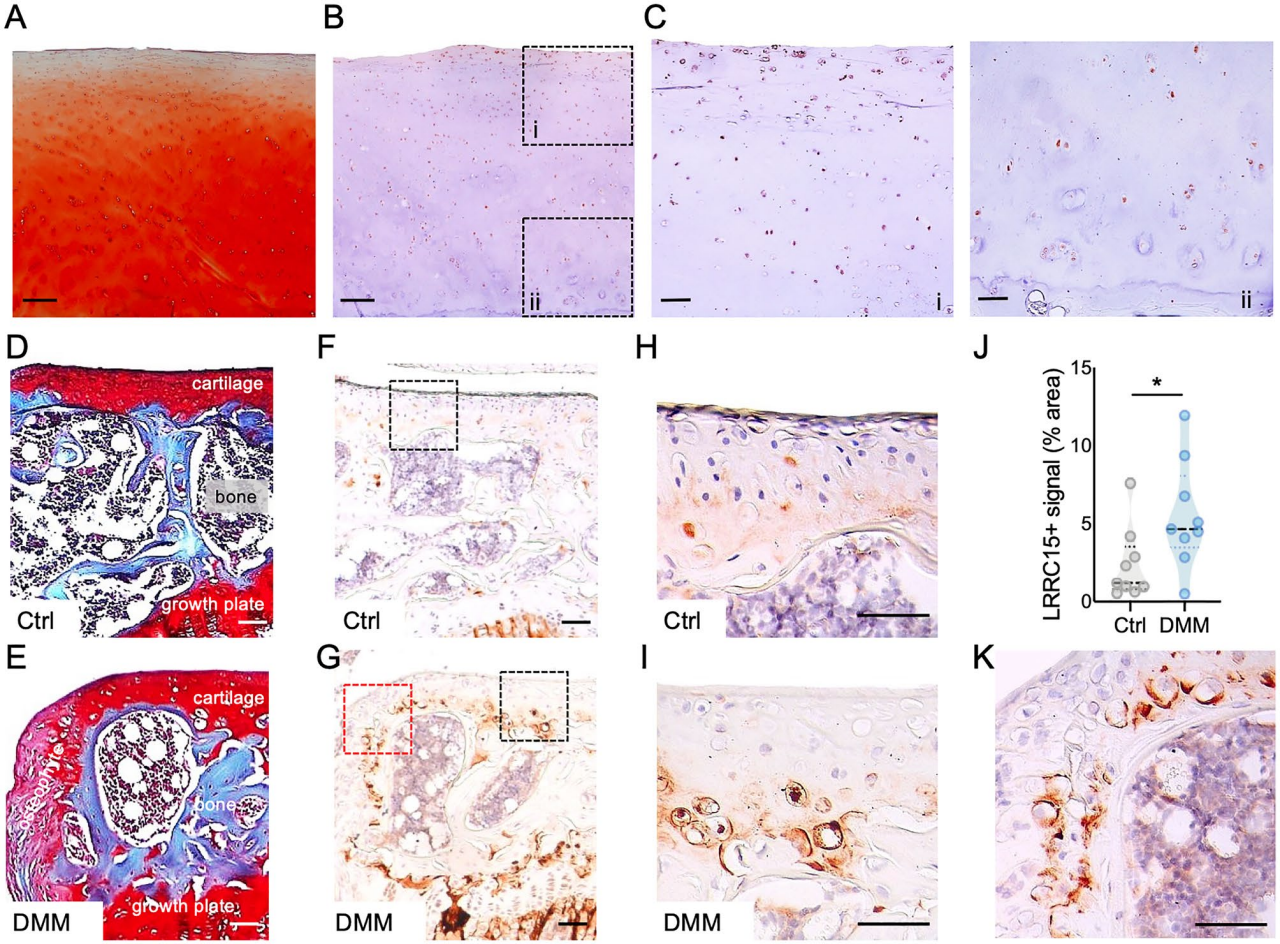
LRRC15 immunostaining in human and mouse OA cartilage. Representative images of (**A**) a Safranin O-stained human OA cartilage specimen, and (**B**) an adjacent section stained with an antibody against LRRC15 (brown indicates positive staining) (4 ×, scale bars = 50 μm). High power view (10 ×) of the (**C**) superficial (upper, i) and deeper (lower, ii) cartilage areas (scale bars = 50 μm). Representative Safranin O-stained (**D**) control (Ctrl) and (**E**) DMM-operated mouse cartilage samples at 4 weeks after DMM (10 ×, scale bars 50 μm). Adjacent (**F**) control and (**G**) DMM-operated sections stained with an antibody against LRRC15 (brown indicates positive staining) (4 ×, scale bars = 50 μm). Dotted areas indicate osteophyte (red) and articular cartilage regions (black) used for higher magnification images. (**H**) Control and (**I**) DMM tissues stained with antibodies against LRRC15 (20 ×, scale bars = 50 μm). (**J**) Quantification of the LRRC15 immunostaining in mouse control (Ctrl) and DMM cartilage tissues (n = 9/ea). *p < 0.05 by t-test. (**K**) Representative LRRC15 immunostaining in the newly formed osteophytes in DMM-operated tissues (20 ×, scale bar = 50 μm).

To further confirm the increased levels of Lrrc15 in OA pathology and its potential functional relevance, we next evaluated the presence of LRRC15 protein in OA cartilage. We retrieved human cartilage samples from patients undergoing total knee replacement for OA (N = 5). Figure 4A shows an example of a Safranin O-stained tissue with relatively intact structure, retaining superficial cartilage. Adjacent serial sections were used for LRRC15 immunostaining, which showed LRCC15 protein distributed throughout all the cartilage zones (Fig. 4B,C and Figure S5). LRRC15 immunostaining was observed in all human OA cartilage samples analyzed, independent of the severity of the structural damage (see Supplementary Figure S5 for additional examples). Similarly, we selected control and DMM-operated mouse knees at 4 weeks after surgery for LRRC15 immunostaining. We stained sections from control (Fig. 4D) and DMM-operated (Fig. 4E) knees with Safranin O/Fast green, and we incubated adjacent sections with anti-LRRC15 antibodies. In agreement with our RNAseq and qPCR data, the DMM-operated tissues showed increased LRRC15 signal relative to control samples (Fig. 4F-I, quantification in Fig. 4J). The increased LRRC15 positive immunostaining was particularly prominent in the deep/calcified cartilage zones in DMM-operated tissues. LRRC15 immunostaining was also very prominent in areas of osteophyte formation in DMM-operated limbs (Fig. 4K), and in the hypertrophic zones in the postnatal growth plates (Supplementary Figure S6). These results confirmed the presence of LRRC15 protein in OA articular cartilage, and further suggested that increased LRRC15 may contribute to disease progression and to changes in OA chondrocyte phenotype and responses.

### *Lrrc15* expression is induced by inflammatory cytokines and DNA demethylation, and contributes to the stress/inflammation-induced gene expression in chondrocytes in vitro

**Figure 5:**
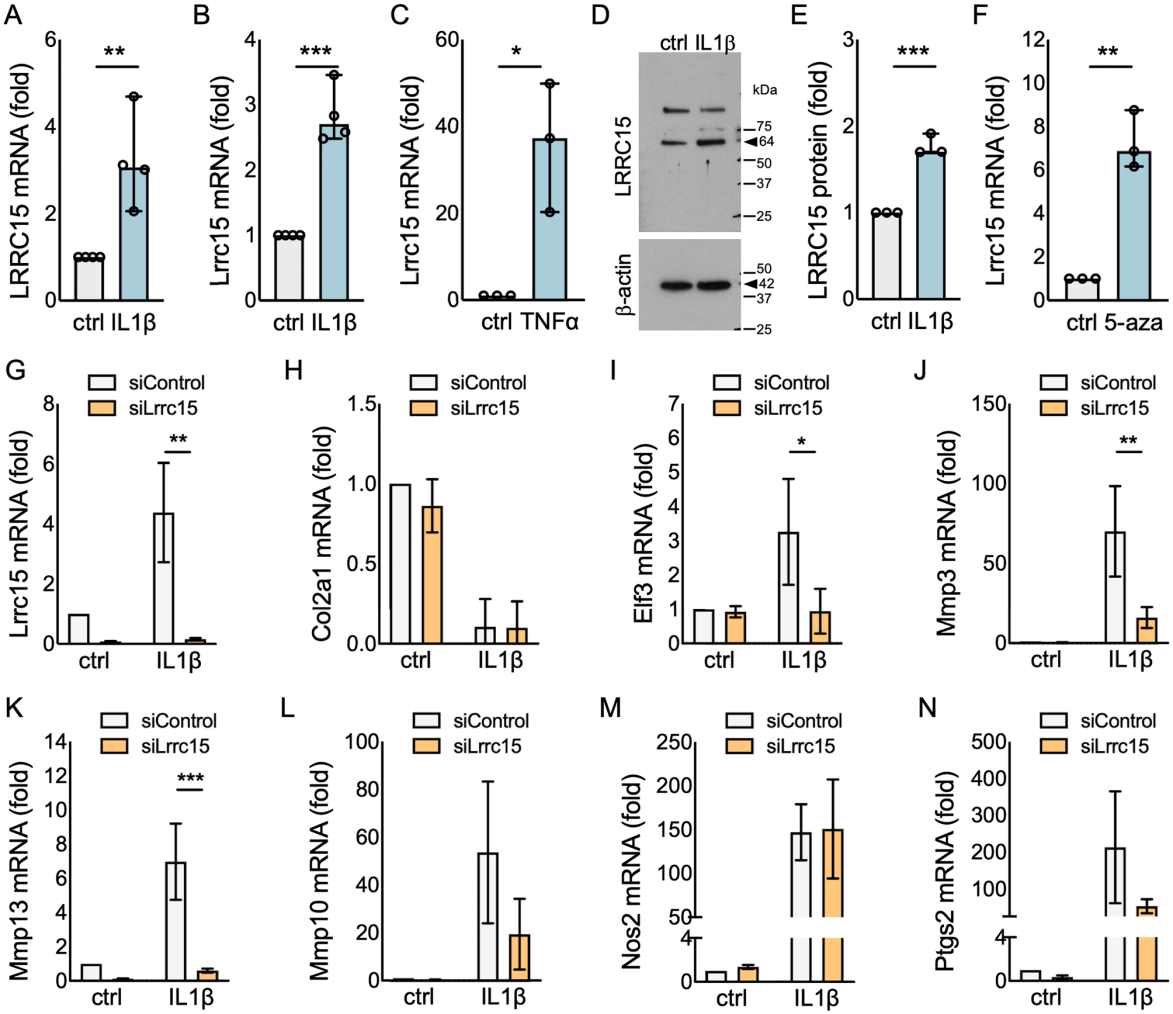
Lrrc15 expression is induced by cytokine stimulation and DNA demethylation, and contributes to the IL-1β-induced gene expression in mouse chondrocytes in vitro. RTqPCR analyses showing (**A**) IL-1β-induced LRRC15 expression in human primary chondrocytes (n = 4), and (**B**) IL-1β-induced (n = 4) and (**C**) TNFα-induced (n = 3) Lrrc15 expression in mouse primary chondrocytes. (**D**) Western blotting analysis of the IL-1β-induced LRRC15 protein in mouse primary chondrocytes. Representative cropped images are shown for clarity and conciseness, and full-length blots are included in the Supplementary Information file. Arrowheads indicate molecular weights for LRRC15 (64 kDa) and β-actin (42 kDa). (**E**) Quantification of the immunoblot (n = 3). (**F**) RTqPCR analyses of mouse chondrocytes (n = 3) treated with 5-Aza-2’-deoxycytidine and trichostatin (labeled as 5-aza) for 72 h, showing increased Lrrc15 expression. Data are shown as fold-change vs. unstimulated controls (set as 1). *p < 0.05, **p < 0.01 and ***p < 0.001 by t-test. RTqPCR analyses in cells transfected with non-targeting control siRNA (siControl) or siRNA against Lrrc15 (siLrrc15), evaluating (**G**) Lrrc15 (**H**) Col2a1, (**I**) Elf3 (**J**) Mmp3, (**K**) Mmp13, (**L**) Mmp10, (**M**) Nos2, and (**N**) Ptgs2 mRNA in cells left untreated (vehicle, ctrl) or treated with 1 ng/ml of IL-1β for 72 h. *p < 0.05, **p < 0.01 and ***p < 0.001 by ANOVA followed by Tukey’s test.

We next investigated changes in *Lrrc15* expression using monolayer cultures of chondrocytes treated with inflammatory cytokines; a model commonly used to mimic OA-like changes *in vitro*^39^. Consistent with other studies^40,41^, IL-1β treatment led to increased *LRRC15* mRNA in human primary chondrocytes (Fig. 5A; see Supplementary Table S7 for RTqPCR primer sequences). We also detected changes in LRRC15 protein in cell lysates from human primary chondrocytes (Supplementary Figure S7). Using murine primary chondrocytes, we confirmed that IL-1β (Fig. 5B) and TNFα (Fig. 5C) treatment induced *Lrrc15* mRNA, and that IL-1β led to increased LRRC15 protein (Fig. 5D,E and Supplementary Figure S8). Long-term treatment with cytokines can produce long-lasting changes in gene expression in chondrocytes, at least in part, by changing DNA methylation patterns^42^. Thus, we tested if long-term stimulation of mouse chondrocytes with IL-1β could lead to sustained increases in *Lrrc15* mRNA expression. As shown in Supplementary Figure S9, murine chondrocytes treated with IL-1β for 2 weeks maintained increased Lrrc15 expression even after passage and 2 additional weeks of culture with cytokine withdrawal. This observation, together with the hypomethylation of the *Lrrc15* gene after DMM (Fig. 3A), suggested that changes in DNA methylation may have a functional impact on *Lrrc15* transcription. To test this, we treated murine primary chondrocytes with the DNA methyl transferase inhibitor, 5-Aza-2’-deoxycytidine (5-aza) combined with the histone deacetylase inhibitor trichostatin (TS), as described^42–44^. We confirmed the expected efficacy of this treatment by assessing changes in *Mmp13* (Supplementary Figure S10)^44^. Evaluation of the Lrrc15 mRNA levels showed an early (72 h, Fig. 5F) and sustained (1-week, Supplementary Figure S10) increase in expression in cells treated with the inhibitor combination, further suggesting that the *Lrrc15* gene transcription in chondrocytes is, at least in part, driven by DNA de-methylation.

Finally, to begin to understand the functional impact of *Lrrc15* in chondrocytes, we tested if *Lrrc15* knockdown (KD) could modify the responses of articular chondrocytes to IL-1β. We first tested the KD efficacy of 3 different custom-designed siRNA oligos against mouse *Lrrc15* (siLrrc15; see Supplementary Table S8 for siRNA oligonucleotide sequences). We selected the siLrrc15 oligo 1 for further experiments because it significantly reduced *Lrrc15* mRNA without impacting *Lrrc17*, whereas the other two oligos showed similar *Lrrc15* knockdown efficacy, but less specificity (Supplementary Figure S11). Next, we transfected primary murine chondrocytes with siControl or siLrrc15 oligos, followed by treatment without or with 1 ng/ml of IL-1β. At 72 h after IL-1β treatment, the siLrrc15 cells displayed reduced *Lrrc15* mRNA compared to siControl cells, and siLrrc15 transfection also blocked the induction by IL-1β treatment (Fig. 5G). While IL-1β-driven repression of *Col2a1* mRNA was not significantly different between siControl and siLrrc15 cells (Fig. 5H), the IL-1β-induced expression of *Elf3* (Fig. 5I), *Mmp3* (Fig. 5J), and *Mmp13* (Fig. 5K) was significantly reduced after *Lrcc15* knockdown. However, we did not detect significant changes in the expression of other genes involved in cartilage catabolism, such as *Mmp10* (Fig. 5L), *Nos2* (Fig. 5M) and *Ptgs2* (Fig. 5N). Together, these results suggest that *Lrrc15* contributes, in a gene-specific manner, to the stress/inflammation-driven expression of genes involved in matrix remodeling and cartilage catabolism in OA.

## Discussion

Dysregulated maintenance of DNA methylation patterns and increased 5hmC levels in articular chondrocytes contribute to the progression of OA^7,8^, but time-dependent changes in 5mC in OA cartilage are relatively unexplored. We used a well-established mouse model of surgically induced post-traumatic OA (PTOA) to capture changes in gene expression and 5mC that occur during the progression of the disease. Our integrative analyses and the comparison with human datasets led to the identification of epigenomic signatures that overlap with changes in gene expression that indicate enrichment of pathways relevant to cartilage development. We also identified differential expression of *Lrrc15* associated with 5mC hypomethylation during the early disease stages. LRRC15 participates in functional networks that include stress responses and extracellular matrix degradative processes, and contributes to the stress/inflammation-induced responses of chondrocytes in vitro.

Our RNAseq data showed the enrichment of genes and functional pathways relevant to cartilage development, hypertrophy, and ossification. This is consistent with previous studies using human and murine cartilage samples, and further reinforces the notion that OA chondrocytes undergo a phenotypic shift and recapitulate developmental steps in an attempt to repair tissue damage (for review, see^2,45–47^). Interestingly, while the enrichment in cell–cell and cell–matrix interactions, hypertrophy, ossification, and ECM assembly pathways are constant, the specific genes up- and down-regulated differ between the 4- and 12-week time-points. This could be a consequence of gene-specific transcriptional kinetics and temporal engagement of different transcriptional networks, but it also suggests that whole-tissue transcriptomic analyses may partly reflect the loss of cartilage structure in more advanced OA disease and, therefore, the loss of specific cellular subsets that are responding to different stimuli and expressing a different array of OA-related genes^28^. More importantly, these time-specific changes highlight the need for developing targeted approaches that take into account disease stage-specific transcriptional changes.

Previous studies showed that changes in DNA methylation are associated with OA disease, and that the maintenance of adequate DNA methylation patterns is required to maintain cartilage homeostasis^5,7,8,30,48^. These studies highlighted that changes in unique CpG sites can impact the transcriptional activity of OA-related genes^49,50^, and that 5hmC accumulation occurs in human OA cartilage^14^ and significantly contributes to the progression of OA^8^. Our RRoxBS data agree with these studies, showing profound changes in 5mC and 5hmC patterns accompanying structural and transcriptional changes during the progression of OA after DMM. Integrating RNAseq and 5mC data, we found that changes in DNA methylation are associated with an enrichment of developmental pathways in OA chondrocytes, consistent with reports indicating that OA chondrocytes recapitulate developmental steps, likely reflecting an early attempt to repair tissue damage. We observed more pronounced changes in 5mC than in 5hmC in our analyses, and showed accumulation of 5hmC following DMM, consistent with recent reports^8^. The less pronounced 5hmC changes observed in our study may be due to the different platforms used to assay and analyze DNA methylation patterns, because RRoxBS selects for GC-rich genomic regions and covers the majority of gene promoters and CpG islands, but provides limited coverage of CpG shores and other relevant intergenic regions^22,51^ that accumulate 5hmC during the progression of OA^8^. These differences notwithstanding, our results provide further evidence of the impact of changes in 5mC to OA and highlight the need of evaluating 5mC/5hmC homeostasis to dissect their relative contribution to the disease.

Integration of our RNAseq and RRoxBS datasets allowed us to identify changes in gene expression associated with changes in DNA methylation patterns following initiation of OA by DMM surgery. Additional bioinformatics comparisons with human data enabled us to uncover clinically relevant targets and changes at the early disease stage. These integrative analyses highlighted *Lrrc15* as one of the genes with increased expression and significant 5mC hypomethylation in early OA cartilage. The *Lrrc15* gene is an evolutionarily conserved leucine rich transmembrane protein^40^, and member of the LRR superfamily^52^. *Lrrc15* expression is restricted to the placental cytotrophoblast during development but upregulated in invasive cancer cells and stimulated by inflammatory cytokines in adult tissues^40,53,54^. Early work showed that Lrrc15 is induced by beta-amyloid and different inflammatory cytokines in rat astrocytes^40^, suggesting that NF-κB and JNK/AP1 signaling are upstream Lrrc15. In addition to inflammatory stimuli, Lrrc15 expression is associated to TGFβ signaling in cancer-associated fibroblasts^53,55^, and these same Lrrc15-positive myofibroblast signatures have been found in arthritic and fibrotic tissues in recent studies using single-cell transcriptomics^56^. Previous reports showed increased LRRC15 mRNA in human OA cartilage and in osteoclasts from patients with rheumatoid arthritis^17,19,31^. Accordingly, we found increased Lrrc15 mRNA and protein levels upon cytokine stimulation of human and murine chondrocytes, and increased LRRC15 immunostaining in OA cartilage. We also observed very prominent LRRC15-positive immunostaining in postnatal growth plates and in developing osteophytes, and our bioinformatics analyses showed that Lrrc15 participates in collagen binding networks and inflammatory signaling. This is in agreement with the proposed role of Lrrc15 in promoting osteogenesis and modulating NF-κB signaling^41^. We found that *Lrrc15* knockdown leads to reduced IL-1β-driven expression of a number of NF-κB targests with known association with OA disease, including *Mmp13* and *Elf3*, whereas other known direct canonical NF-κB targets, such as *Nos2* and *Ptgs2*^45^, are not affected by the *Lrrc15* knockdown. Thus, it is conceivable that *Lrrc15* drives gene expression in a cell- and gene-specific context, likely via concerted modulation of canonical NF-κB and other signaling pathways. In keeping with these observations, ingenuity pathway analyses to identify molecular interactions predicted that Lrrc15 belongs to molecular regulatory pathways interacting with transcriptional regulators and cytokines with known roles in stress/inflammatory signaling and apoptosis (see results). A greater mechanistic understanding of the signaling events upstream and downstream Lrrc15 is required to dissect the contribution of Lrrc15 to OA disease, including experiments with enforced expression of exogenous Lrrc15.

Taken together, our data suggest that increased *Lrrc15* levels in early OA may represent an early event in the chondrocyte activation characteristic of OA, which may in turn contribute to disease progression and to permanent changes in OA chondrocyte phenotype and responses in an attempt to repair tissue damage recapitulating developmental processes. We believe that Lrrc15 may contribute to these functional and phenotypic alterations of chondrocytes by coordinating stress responses and dysregulated extracular matrix remodeling. The contribution of *Lrrc15* to signaling pathways involved in regulation of cartilage damage and chondrocyte function should be further investigated.

Our study has limitations. The integration of our datasets with human orthologs using HuGENet confirmed the utility of the DMM model as a preclinical exploratory tool and identified conserved OA-related changes in gene expression and DNA methylation. However, whole tissue transcriptomic and epigenomic analyses do not account for the cellular heterogeneity and spatial variability. Integrated single-cell and spatial transcriptomics and epigenomics analyses could provide a better understanding of specific targets during disease progression. In addition, while the comparisons between non-operated and operated joints yielded results that are consistent with the literature, the lack of sham or naïve controls from separate mice is a limitation. The use of male mice is another limitation, and follow-up studies should include female mice.

Overall, our study provides new insights about the contribution of 5mC changes to cartilage damage in OA, and highlights *Lrrc15* as a novel gene with potential contribution to the functional and phenotypic changes of OA chondrocytes, coordinating stress responses and altered ECM remodeling. Studies that further address the functional impact of these changes in vivo are required and may provide insight to develop targeted preventative therapies.

## Methods

See Supplementary Information for a detailed outline of the methods, procedures and specific materials used in this study.

### Ethics statement

All experiments were performed according to the guidelines of the American Veterinary Association and were approved by the IACUC of the Hospital for Special Surgery, and all procedures are reported following the ARRIVE guidelines^57^. Experiments conducted using human cells and cartilage were done with approval by the Institutional Review Board (IRB) of the Hospital for Special Surgery (HSS). De-identified cartilage samples were retrieved at the time of total knee replacement surgery for OA after written informed consent was obtained, and the study and all methods were performed in accordance with the relevant guidelines and regulations.

### Destabilization of the medial meniscus (DMM) surgery and tissue processing post-DMM

DMM surgeries were performed in 12-week-old male C57BL/6J (N = 53) purchased from the Jackson Laboratory (Bar Harbor, ME, USA), as described^15,16^. Mice were housed 5 per cage and allowed to acclimate for two weeks before experiments. Food and water were provided ad libitum. Power analyses were performed to calculate sample size for the surgical experiments. The left knees were unoperated and served as contralateral controls. Animals were euthanized at 4 weeks after surgery (n = 9 for histology and immunohistochemistry, n = 9 for RNA isolation and n = 9 for DNA isolation) or at 12 weeks after surgery (n = 8 for histology and immunohistochemistry, n = 9 for RNA isolation and n = 9 for DNA isolation). For RNA and DNA isolation, 3 microdissected cartilage samples were pooled together to obtain 1 RNA or 1 DNA sample for RNA-seq or RRoxBS analyses, respectively. Knees were collected and processed for histological scoring of OA pathology as assessed by two blinded scorers^15,58^. Briefly, coronal sections of 6 μm were cut across the whole joint, deparaffinized in xylene, rehydrated through an ethanol series, and stained with Safranin O/Fast green. OARSI SUM scores were obtained by grading 8 sections per knee joint (each data point represents the summed scores of 8 sections/mouse), essentially as described^15,16^. Adjacent sections were used for immunohistochemical analyses.

### Reduced representation oxidative bisulfite sequencing (RRoxBS)

Modifications in 5-methylcytosine (5mC) and 5-hydroxymethylcytosine (5hmC) were determined using oxidative bisulfite sequencing (oxBS-Seq) in combination with reduced representation bisulfite sequencing (RRBS)^21,22^. Briefly, ~ 200 ng of genomic DNA were digested with 100U of MspI (New England Biolabs, Ipswich, MA) and purified using QIAquick PCR purification columns (Hilden, Germany). The digested DNA was spiked with 0.01% control DNA duplexes, provided by Cambridge Epigenetix (Cambridge, UK), containing C, 5mC and 5hmC bases at known positions. The control sequences were later analyzed after sequencing to give a quantitative assessment of the efficiency of oxidation and bisulfite conversion. Six uniquely indexed libraries were generated using the Ovation Ultralow Methyl-Seq DR Multiplex with TrueMethyl oxBS kit and workflow (Tecan, Redwood, CA). Before oxidation and bisulfite-oxidation treatment, the six libraries were combined and concentrated over a QIAquick PCR purification column (Hilden, Germany) to obtain one pool. This library pool was split into two equal aliquots: one aliquot was oxidized, and bisulfite converted (oxBS), the other one subjected to a mock oxidation before bisulfite conversion (BS). A total of 2 μl from each of the pools was assessed by qPCR to determine the optimal number of PCR amplification cycles to obtain enough material for sequencing. The BS library pool was amplified with 7 cycles and the oxBS library pool for 9 cycles. After amplification, each library pool was normalized to 2 nM and independently clustered at 6.5 pM on single read flow cell and sequenced for 50 cycles on an Illumina HiSeq 2500. Illumina’s CASAVA 2.17 software pipeline was used to perform image capture, base calling, and demultiplexing of raw reads to produce FASTQ files. Quality of the raw sequence reads was assessed with FastQC (Version 0.10, Babraham Bioinformatics) and FASTX toolkit (version 0.0.13, http://hannonlab.cshl.edu/fastx_toolkit/). Raw reads were then adapter trimmed, aligned, and post-processed to produce methylation calls at base pair resolution using a previously described in-house pipeline using CUTADAPT instead of FLEXBAR to trim the reads^59^.

### Differential methylation analysis

CpG sites from the resulting RRoxBS data were interrogated for true methylation (5mC) and hydroxy methylation (5hmC), by subtracting the oxBS methylation call from the BS methylation call on the same CpG, and for differential methylation (q value < 0.05 and methylation percentage difference of at least 25%) using methylKit^60^. The differentially methylated (5mC) CpG (DMC) sites were then queried for differentially methylated regions (DMRs) using eDMR^61^. A DMR was defined as a 100 bp genomic region with at least 3 CpGs, with at least 1 CpG significantly differentially methylated (25% methylation difference, q < 0.01) and an overall average differential methylation of at least 20% across all the CpGs. DMRs were annotated for proximal genes. Downstream statistical analyses and plots were generated using the R software environment for statistical computing (https://www.r-project.org/).

### Low input RNAseq

Libraries for RNAseq were prepared from 3 ng of RNA, by first generating full length double stranded cDNA (ds cDNA) using the SMART-Seq v4 Ultra™ Low Input kit (Cat. 634891, Takara Bio USA) followed by Illumina’s Nextera XT DNA Library Preparation kit (FC-131–1096, Illumina Inc. San Diego, CA). Briefly, 3 ng of RNA were used to obtain first strand cDNA using SMART-Seq2 template switching and extension with SMARTScribe™ reverse transcriptase. cDNA was amplified using 10 cycles of PCR with SeqAmp DNA polymerase. The resulting dscDNA was validated by determining average size (~ 2 kb) on an Agilent Bioanalyzer high sensitivity chip. 500 pg of the dscDNA were tagmented with the Nextera transposome to fragments of ~ 350 bp containing adapter sequences. Unique Indexes for each library were added by using 12 cycles of PCR amplification and resulting libraries were pooled together for sequencing. The pools were clustered at 6.5 pM on single read flow cell and sequenced for 50 cycles on an Illumina HiSeq 2500. Base call files generated from the sequencer were demultiplexed and converted to FASTQ files using the Illumina CASAVA 2.17 pipeline. Quality of the raw sequence reads was assessed with FastQC (Version 0.10, Babraham Bioinformatics) and FASTX toolkit (version 0.0.13, http://hannonlab.cshl.edu/fastx_toolkit/).

### Differential expression analysis

RNAseq reads passing Illumina’s purity filter were adapter trimmed using FLEXBAR barcode and adapter processing tool^62^. The trimmed reads were aligned to the Gencode (https://www.gencodegenes.org/) mm10/ GRC38.p3 build of the mouse genome using STAR aligner with default parameters^63^. Read counts for RefSeq (NCBI) transcripts were then quantified from the alignments using the featureCounts software package^64^. Significant differential expression (adjusted p-value < 0.05) was assessed between the read counts for the control and DMM sample groups using the DESeq2 R Bioconductor package^65^.

### Functional interpretation

Gene Ontology (GO) enrichment (pAdjustMethod = “BH”, pvalue Cutoff = 0.01, qvalue Cutoff = 0.05) analysis for Biological Processes (BP), Cellular Components (CC) and Molecular Functions (MF) was performed for differentially expressed genes and genes associated with differentially methylated regions using the clusterProfiler Bioconductor R package^66^. Regulatory networks of differentially expressed and/or methylated genes were generated through the use of through the use of IPA (QIAGEN Inc., https://www.qiagenbioinformatics.com/products/ingenuity-pathway-analysis). Downstream statistical analyses and plots were generated using the R software environment for statistical computing.

### Comparison with human datasets

Using human orthologs (assigned via biomaRt R interface), genes that had differential expression (DEGs) and/or differential methylation (DMRs) associated with them were compared to genes known to be affected in human OA studies using HuGENet and 10 published datasets, including 4 datasets comparing OA cartilage samples with healthy controls^17,31–33^ and 6 comparing eroded OA cartilage or subchondral bone with adjacent non-eroded controls^6,18,25,26,29,30^. VennDiagram (R package) was used to look at intersections of HuGENet with DEGs and DMRs significant in our study. Likewise, VennDiagram and UpSetR (R packages) were used to represent intersections with DEGs and DMRs between human OA data and datasets generated from this study.

### Statistical analysis

Statistical analyses of the RTqPCR data and histological scores were performed using GraphPad Prism 8 Software (GraphPad Software, San Diego, CA). Data are reported as means ± S.D. or as median and 95% C.I. (histological scores) of at least three independent experiments. For in vitro experiments, each data point represents one independent experiment performed using primary chondrocytes isolated from articular cartilage retrieved from a different OA patient (human) or mouse pup (murine). Unpaired Student t-test was used to establish statistical significance between two groups. Analysis of the histological scores was performed using the Mann–Whitney test. For data involving multiple groups, one-way analysis of variance (ANOVA) was performed, followed by Tukey’s post-hoc test. P < 0.05 was considered significant.

## Supplementary Information

Supplementary Information 1.

Supplementary Table S1.

Supplementary Table S2.

Supplementary Table S3.

Supplementary Table S4.

Supplementary Table S5.

Supplementary Table S6.

## Data Availability

The data that supports the findings of this study are available from the corresponding author on reasonable request. RNAseq and RRoxBS sequencing data have been deposited at the GEO database with accession code GSE175486.
