# Supplementary material for: Transcriptomic and epigenomic analyses uncovered Lrrc15 as a contributing factor to cartilage damage in osteoarthritis
Source: Sci Rep. 2021 Oct 26;11:21107. doi: 10.1038/s41598-021-00269-8 (PMC8548547; doi:10.1038/s41598-021-00269-8)
Supplement: Supplementary file 1 — Supplementary Information 1. [file 41598_2021_269_MOESM1_ESM.pdf]

Supplementary Materials for

Transcriptomic and epigenomic analyses uncovered Lrrc15 as a contributing factor to cartilage damage in osteoarthritis.

Purva Singh[1†], Mengying Wang[1,2†], Piali Mukherjee[3], Samantha G. Lessard[1], Tania Pannellini[1], Camila B. Carballo[1], Scott A. Rodeo[1,3], Mary B. Goldring[1,3], and Miguel Otero[1,3*]

[1]HSS Research Institute, Hospital for Special Surgery, New York, NY, 10021, USA. [2]School of Public Health, Xi'an Jiaotong University Health Science Center, Xi'an, China. [3]Weill Cornell Medical College, New York, NY, 10021, USA.

[†]Purva Singh and Mengying Wang contributed equally

[*]To whom correspondence should be addressed: Miguel Otero, Ph.D., Hospital for Special Surgery, HSS Research Institute, Orthopedic Soft Tissue Research Program, Room 603, 515 East 71st Street, New York, NY 10021, USA; Tel. 212-774-7561; Fax. 617-249-2373; E-mail: OteroM@hss.edu

This file includes:

Supplementary Materials and Methods
Supplementary References
Figure S1: Common differentially expressed genes (DEGs) at 4 and 12 weeks after DMM.
Figure S2: Significantly enriched Gene Ontology categories based on differential gene expression.
Figure S3: Changes in DNA methylation in cartilage following DMM surgery.
Figure S4: Comparison of RNAseq and RRxoBS data after DMM with published human datasets.
Figure S5: LRRC15 immunostaining in human OA cartilage samples
Figure S6: LRRC15 immunostaining in mouse postnatal growth plates
Figure S7: Increased LRRC15 protein in human primary chondrocytes after IL1β treatment.
Figure S8: Increased LRRC15 protein in mouse primary chondrocytes after IL1β treatment.
Figure S9: Long-term cytokine treatment leads to sustained increased Lrrc15 mRNA levels in vitro.
Figure S10: Treatment with DNMT inhibitors leads to sustained (1 week) increased Lrrc15 mRNA.
Figure S11: Evaluation of the Lrrc15 knockdown in cells transfected with custom siRNA oligos.
Table S7: qPCR primers and conditions.
Table S8: Custom siRNA oligonucleotide sequences.

Other Supplementary Materials for this manuscript include the following:

Table S1: Differentially expressed genes in microdissected cartilage at 4 and 12 weeks after DMM.
Table S2: GO categories using RNAseq.
Table S3: Significantly methylated 5mCs and 5hmCs after DMM.
Table S4: Significant differentially methylated regions (DMR) after DMM.
Table S5: GO categories integrating differential expression and methylation.
Table S6: Summary of comparative analyses against human datasets.

MATERIALS AND METHODS

RNA isolation from micro-dissected mouse cartilage

We extracted total RNA from micro-dissected articular cartilage of control or DMM-operated knees, essentially as described[1]. Briefly, articular cartilage was removed from the tibial plateaus and femoral condyles with a scalpel blade while bathed in RNA later (Ambion) under a dissection microscope. Total RNA was extracted using TRIzol combined with the RNeasy mini kit protocol (Qiagen). Microdissected cartilage tissues from 3 controls or 3 DMM-operated knees were pooled to generate one sample. After pooling, tissues were homogenized in TRIzol using a TissueLyser (Qiagen) set at 300Hz for 2min, followed by QIAshredder (Qiagen). The supernatant was mixed with chloroform: isoamyl alcohol (24:1), incubated on ice for 5 min and centrifuged for separation of phases for 10 min. The separated aqueous phase was mixed with 1.25 times 100% ethanol and subjected to RNA purification and DNase treatment following the RNeasy mini kit instructions. RNA integrity was assessed at the Core Laboratories Center of Weill Cornell Medicine (WCM). For RNAseq and RTqPCR analyses, total RNA with RIN>7 and 260/280>1.8 was used.

DNA isolation from micro-dissected mouse cartilage

Microdissected cartilage tissues from 3 controls or 3 DMM-operated knees were pooled to generate one sample. Pooled cartilage samples were homogenized in 1X PBS using a 1.5ml pestle and a handheld motorized homogenizer (Argos, Catalog No A0001). DNA was isolated using the Gentra Puregene DNA isolation kit (Qiagen) protocol with minor modifications, as described[1]. Briefly, homogenized cartilage was lysed in lysis buffer supplemented with proteinase K. Lysed samples were then incubated with RNase for 45 min at 37°C, followed by protein precipitation. For DNA precipitation, samples were incubated in isopropanol -20°C for 90 minutes, centrifuged at 13,000 xg for 10 min, and washed 3 times in 70% ethanol. Finally, DNA pellets were reconstituted in DNA hybridization buffer. Only DNA with a 260/280>1.8 was used for RRoxBS analyses. Additional quality control steps were done at the WCM Epigenomics Core.

Chondrocyte isolation and culture, and RNA isolation

Human chondrocytes were isolated by sequential digestion with Pronase (Promega) and collagenase P (Promega), as described[2], from the articular cartilage obtained from osteoarthritis patients undergoing total knee replacement surgery, with approval by the Institutional Review Board (IRB) of the Hospital for Special Surgery, and patient consent. After isolation, human primary chondrocytes were plated in complete medium (DMEM/F12 + 10% FBS + 1% Pen/Strep) at a density of $5x10^4$ cells/cm^2 until confluence and used at passage 1 for experiments. Mouse primary chondrocytes were isolated from 5- to 6-day old C57BL/6J mice by sequential digestion with Collagenase D, also as described[3]. Murine cells were plated

at a density of $15x10^3$ cells/cm^2 , expanded in complete medium and used for experiments between passages 2 and 3. Experiments involving cytokine or 5-Aza-2'-deoxycytidine and trichostatin treatment were conducted as previously described[4,5], using IL-1β (1ng/ml), TNFα (10 ng/ml), or a combination of 5- Aza-2'-deoxycytidine (5-aza, 2uM) and trichostatin (TS, 40nM) for the indicated time-points. For total RNA isolation, cells were lysed in RLT buffer containing 2-mercaptoethanol, and total RNA was isolated using the RNeasy miniRNA isolation kit, following the manufacturer's instructions (Qiagen).

Quantitative reverse transcription PCR (RT-qPCR)

Total RNA was reversed transcribed using the iscript2 reverse transcriptase kit (Bio-Rad). Gene amplifications were carried out using SYBR Green I-based real-time PCR, as described[5], using specific primers against *Col2a1*, *Eef1a1*, *Elf3*, *Hprt1*, *Lrrc15*, *Lrrc17*, *Mmp3*, *Mmp10*, *Mmp13*, *Nos2* and *Ptgs2* (Supplementary Table S7). The data were calculated as the ratio of each gene to Hprt1 using the $2^{-\Delta\Delta Ct}$ method for relative quantification. *Eef1a1* was used as an additional housekeeping gene in control experiments, but not used as a normalizer in the final analyses.

Immunoblotting

Monolayer cultures of human and mouse primary chondrocytes were left untreated (control, vehicle) or treated with 1ng/ml of IL-1β for 72 h. After treatment, cells were lysed using lysis buffer containing 50mM Tris-HCl (pH 7.7), 1%Triton X-100, 150mM NaCl, 1mM EDTA, 1mM 1,10-phenanthroline, 1X protease inhibitor, 10mM NaF, 2mM $NaVO_3$, and supplemented with protease inhibitor cocktail (Roche Diagnostics, Indianapolis, IN, USA). Total protein concentration was determined using a BCA protein assay (Pierce Chemical Co., Rockford, IL, USA), and equal concentrations of whole cell lysates were separated by SDS-PAGE in reduced conditions, transferred to PVDF membranes and incubated with primary antibodies against LRRC15 (1:1000, Abcam # ab150376 for mouse samples, Abcam # ab157484 for human samples) and β-actin (1:1000, Sigma # A5441), used as loading control. Blots were developed using the Super Signal West Pico PLUS Chemiluminescent substrate (Thermo Scientific, Pierce Chemical Co., Rockford, IL, USA), and the relative abundance of the LRRC15 protein was determined by densitometry, using ImageJ software (NIH, Bethesda, MD), and normalized to β-actin, as previously described.

LRRC-15 immunohistochemistry (IHC)

Immunostaining was performed in human OA cartilage samples collected at the time of total knee arthroplasty (N=5), under IRB approval and patient consent, and in control and DMM (OA) tissues obtained at 4 weeks post-DMM (N=9/ea) using the Vectastain ABC rabbit IgG kit (Vector Laboratories). Briefly, sections were deparaffinized in xylene and rehydrated in an ethanol series. After quenching of endogenous

peroxidases (3% hydrogen peroxide in PBS for 30 min at room temperature), antigen retrieval (0.05% trypsin and 0.05% $CaCl_2$ in water (w/v) pH 7.8, for 20 min at 37°C), and blocking in protein block for 30 min (Dako), sections were incubated overnight with a rabbit polyclonal LRRC-15 antibody (1:100, Abcam # ab157484). The signal was developed using Nova RED (Vector Laboratories). For negative controls, normal rabbit IgG (Santa Cruz) was used in place of the primary antibody. Digital images of the LRRC15-stained sections were obtained using an upright microscope (Nikon Eclipse 50i) and the image processing Software NIS Elements 4.0. Mouse images were processed using Image J software for relative quantification of the LRRC15 positive signal in control and DMM-operated sections, as described[6].

siRNA transfection and *Lrrc15* knockdown

For knockdown experiments, passage 2 mouse primary chondrocytes were plated in complete medium at a density of 20,000 cells/cm^2 in 6-well culture plates. After 24 h, cells were transfected with either 25nM of mouse *Lrrc15* siRNA (siLrrc15; siGENOME Custom order, Supplementary Table S8) or 25nM of control siRNA (Thermo Scientific, Pittsburgh, PA, USA) in antibiotic-free conditions, using the DharmaFECT transfection reagents following the manufacturer's instructions (ThermoFisher). At 6 hours after transfection, cells were supplemented with 1ml of complete medium without antibiotics. At 24 hours after transfection, the transfection medium was replaced with fresh complete medium for additional 24 hours. Assessment of the *Lrrc15* knockdown efficacy using the 3 different siLrrc15 oligos was done by RTqPCR at 48 hours after transfection. For experiments with cytokines, at 48 hours after transfection cells were left untreated (vehicle) or treated with IL-1β (1ng/ml) for 72 hours, and RNA was isolated as described above.

REFERENCES:

1 Culley, K. L. *et al.* Mouse Models of Osteoarthritis: Surgical Model of Post-traumatic Osteoarthritis Induced by Destabilization of the Medial Meniscus. *Methods Mol Biol* **2221**, 223-260, doi:10.1007/978-1-0716-0989-7_14 (2021).

2 Otero, M. *et al.* Human chondrocyte cultures as models of cartilage-specific gene regulation. *Methods Mol Biol* **806**, 301-336, doi:10.1007/978-1-61779-367-7_21 (2012).

3 Gosset, M., Berenbaum, F., Thirion, S. & Jacques, C. Primary culture and phenotyping of murine chondrocytes. *Nat Protoc* **3**, 1253-1260, doi:10.1038/nprot.2008.95 (2008).

4 Hashimoto, K., Oreffo, R. O., Gibson, M. B., Goldring, M. B. & Roach, H. I. DNA demethylation at specific CpG sites in the IL1B promoter in response to inflammatory cytokines in human articular chondrocytes. *Arthritis Rheum* **60**, 3303-3313, doi:10.1002/art.24882 (2009).

5 Singh, P., Lessard, S. G., Mukherjee, P., Rourke, B. & Otero, M. Changes in DNA methylation accompany changes in gene expression during chondrocyte hypertrophic differentiation in vitro. *Ann N Y Acad Sci*, doi:10.1111/nyas.14494 (2020).

6 Wang, M. *et al.* Knee fibrosis is associated with the development of osteoarthritis in a murine model of tibial compression. *Journal of Orthopaedic Research* **39**, 1030-1040, doi:https://doi.org/10.1002/jor.24815 (2021).

SUPPLEMENTARY FIGURES

Supplementary Figure S1: ***Common differentially expressed genes at 4 and 12 weeks after DMM.*** (**A**) Venn Diagram representation of the unique and overlapping differentially expressed genes at 4 and 12 weeks after DMM, and (**B**) Heatmap representation of the differentially expressed genes that are common to 4 and 12 weeks after DMM. Each row represents one gene, each column represents one time-point. Blue dotted line indicates baseline, with upregulated genes labeled in red and with the blue line to the right.

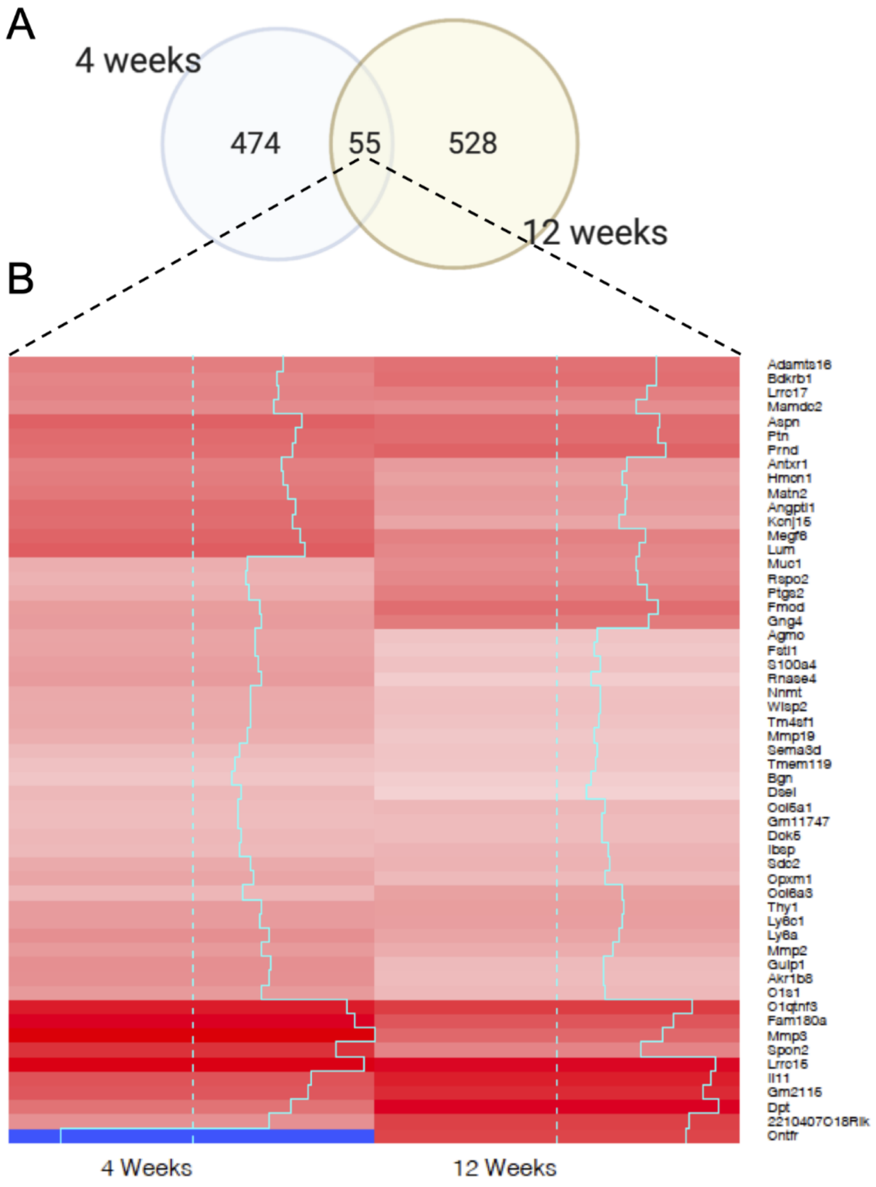

Supplementary Figure S2: Significantly enriched (FDR < 0.05) Gene Ontology categories based on significant differential expression. BP indicates Biological Processes, CC indicates Cellular Components, and MF indicates Molecular Functions.

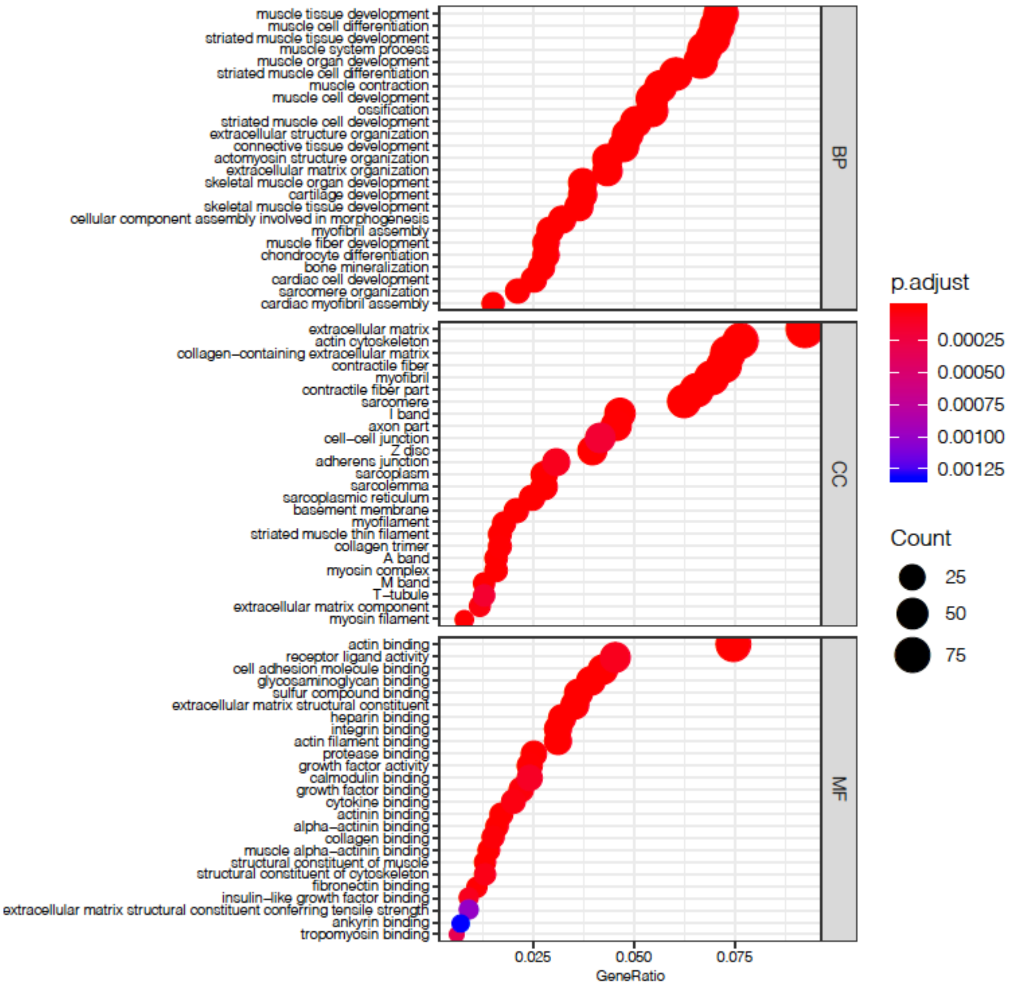

Supplementary Figure S3: Changes in DNA methylation in cartilage following DMM surgery. Representation of the differential hyper- and hypo-methylation (%) in microdissected cartilage from DMM-operated (right surgical limbs, RS) relative to the non-operated (left non-surgical controls, LC) limbs (n=3 per group and time-point). Differential methylation per chromosome plots for 5mC at (**A**) 4 weeks and (**B**) 12 weeks after DMM. Differential methylation per chromosome plots for 5hmC at (**C**) 4 weeks and (**D**) 12 weeks after DMM. Differential methylation was defined as a q value < 0.05 and methylation percentage difference of at least 25%. (**E**) Venn diagram representation of the overlapping 5hmCs for 4 and 12 weeks.

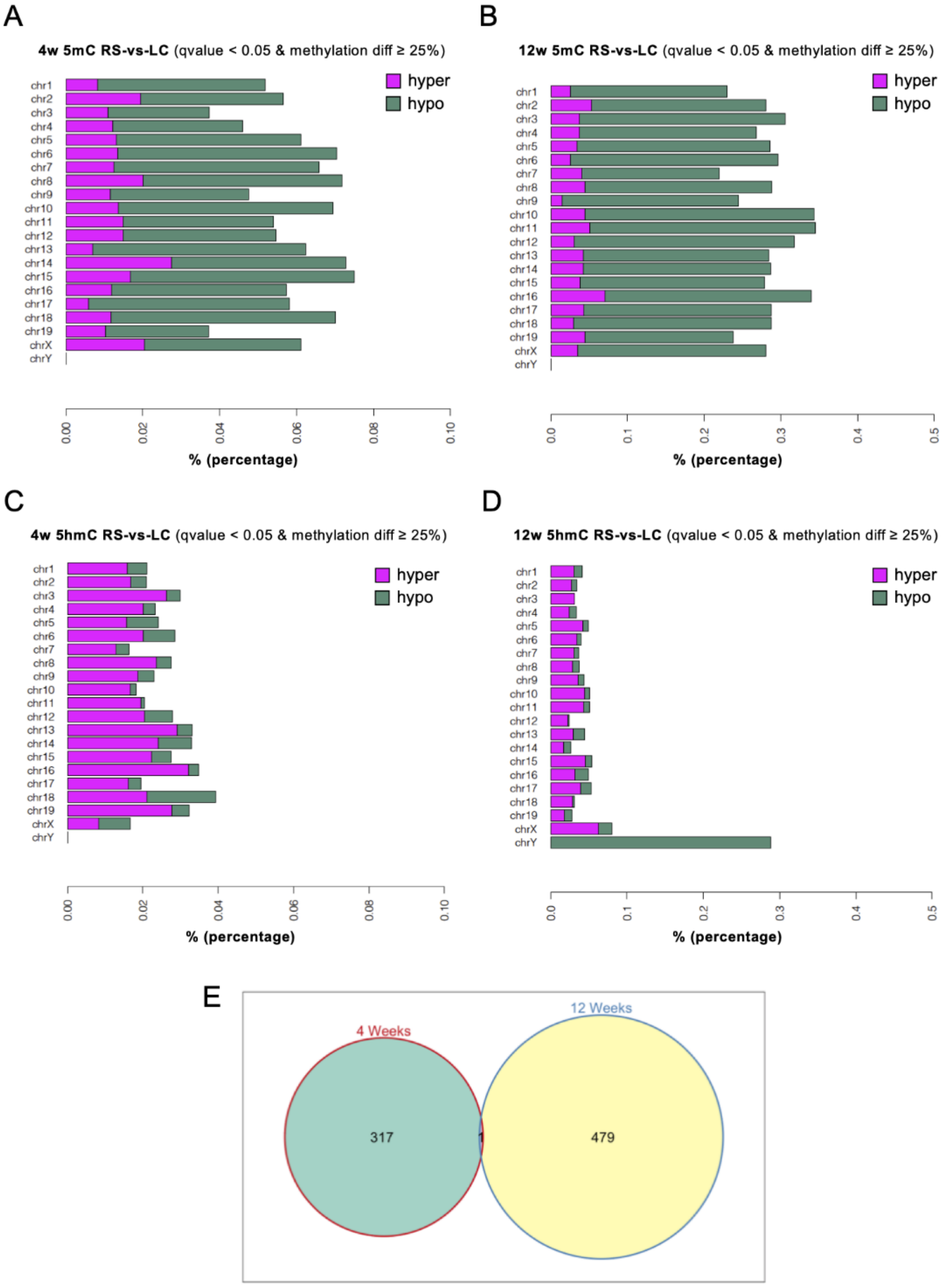

Figure S4: Comparison of RNAseq and RRxoBS data after DMM with published human datasets. UpSet plot representation of the intersections of our dataset (DEGs and DMRs in microdissected cartilage after surgery, 4 and 12 weeks) with selected human datasets including (**A**) eroded vs. non-eroded OA articular cartilage and (**B**) healthy vs. OA cartilage. (**C**) Venn diagram representation of the intersects between HuGENet knee OA genes and DEGs and DMRs significant in our dataset. The gene at the intersection of DEGs, DMRs and HuGENet is *Aspn*.

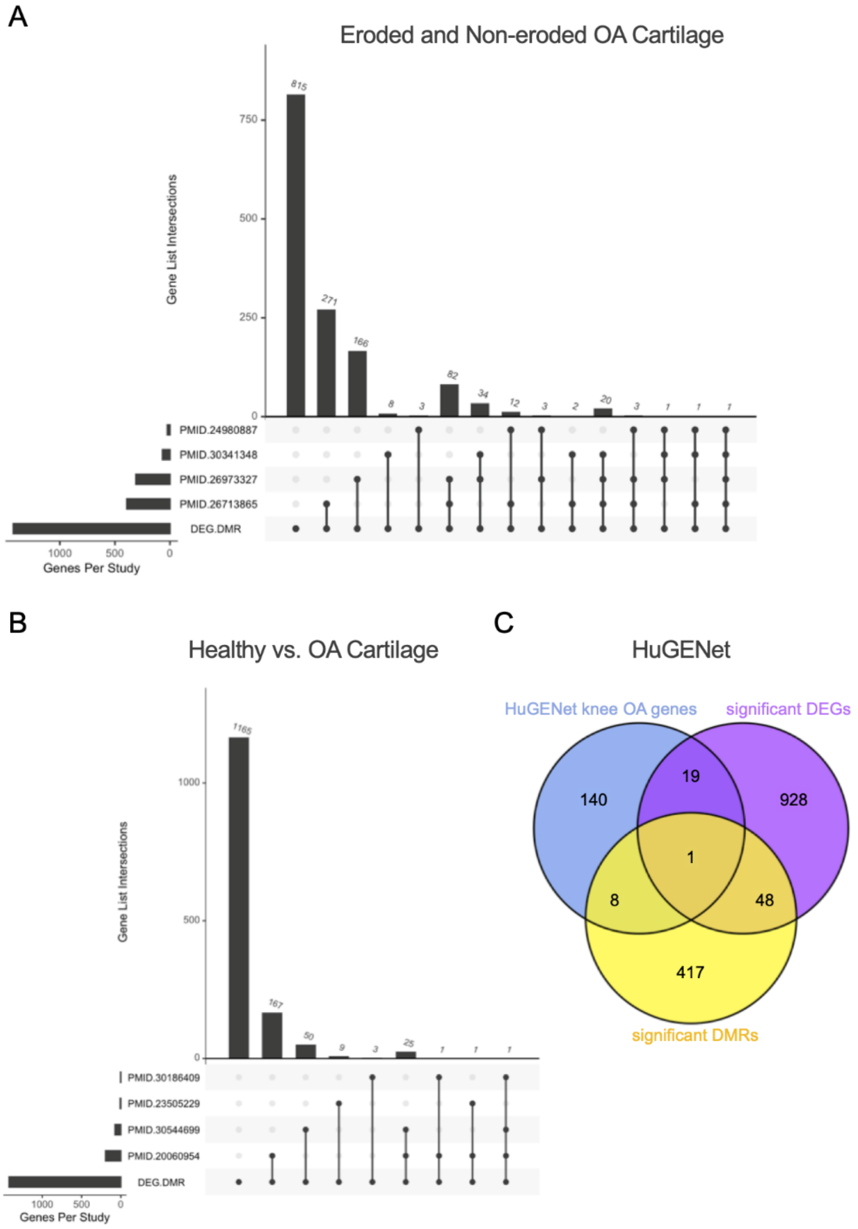

Supplementary Figure S5. LRRC15 immunostaining in human OA cartilage samples. Representative microphotographs of human articular cartilage retrieved from two patients at the time of total knee arthroplasty for osteoarthritis are shown in (**A**) and (**B**). Safranin O-stained sections are shown in (**a.1**) and (**b.1**). Serial adjacent section stained with specific antibodies against LRRC15 are shown in (**a.2**) and (**b.2**). Original magnification 5X. High power view (10X) of two regions of interest (superficial and deep) are shown in (**a.3**), (**a.4**), (**b.3**) and (**b.4**). Brown (arrowheads) indicates positive staining. (**C**) Negative control using normal rabbit IgG instead of primary antibody. Scale bars = 50 µm. (D) Relative quantification of the LRRC15 positive immunostaining comparing regions of interest selected in the superficial and deeper cartilage zones of 3 different patient samples. Data are shown as mean ± standard deviation (error bars).

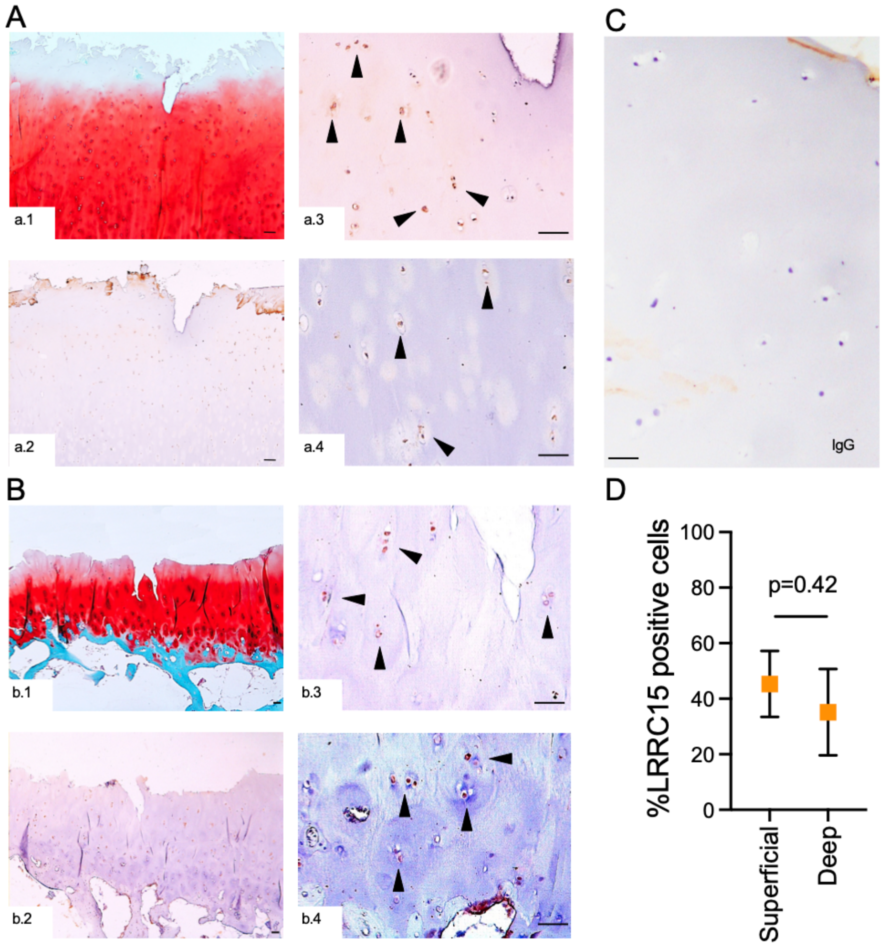

Supplementary Figure S6. LRRC15 immunostaining in mouse limbs. (A) Representative image (original magnification, 4X) showing a mouse limb at 4 weeks after DMM surgery stained with specific antibodies against LRRC15, showing positive immunostaining (brown signal) in the articular cartilage, areas of newly formed osteophytes and postnatal growth plates. **(B)** A selected area of the growth plate (squared in A) is shown in higher magnification (10X). **(C)** Negative control slide (original magnification 20X), using normal rabbit IgG instead of primary antibody. Scale bars = 50 µm

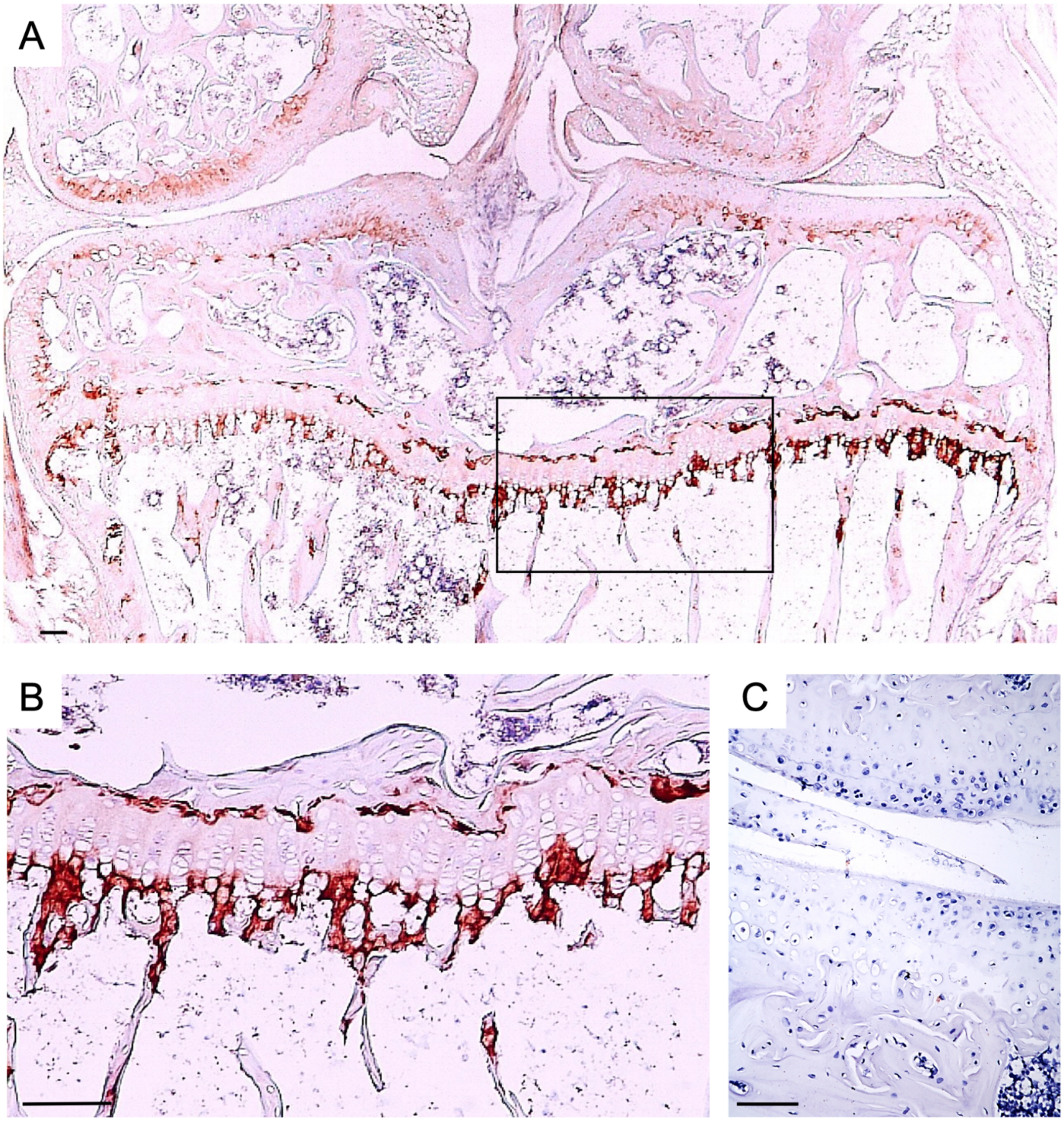

Supplementary Figure S7. Increased LRRC15 protein in human primary chondrocytes after IL1β treatment. Western blotting analysis of the IL-1β-induced LRRC15 protein in passage 1 human primary chondrocytes left untreated (vehicle, 0) or treated with 1 or 10 ng/ml of IL-1β for 72h.

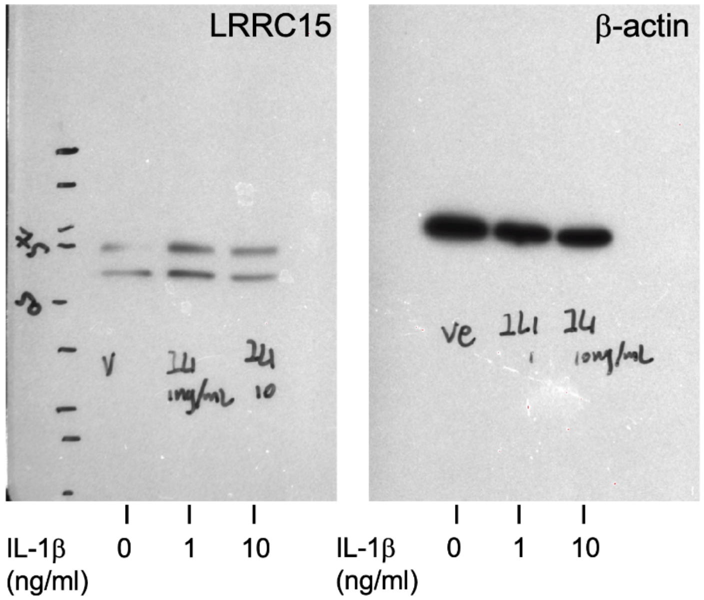

Supplementary Figure S8. Increased LRRC15 protein in mouse primary chondrocytes after IL1β treatment. Western blotting analysis of the IL-1β-induced LRRC15 protein in passage 1 mouse primary chondrocytes left untreated (vehicle, ctrl) or treated with 1 ng/ml of IL-1β for 72h. Two independent experiments performed using cells obtained from different mice are shown, labeled as expm#1 and expm#2. Cropped images of experiment #1 (expm#1) are shown in Figure 5D. Arrowheads indicate the expected molecular weights for LRRC15 (64kDa) and β-actin (42kDa).

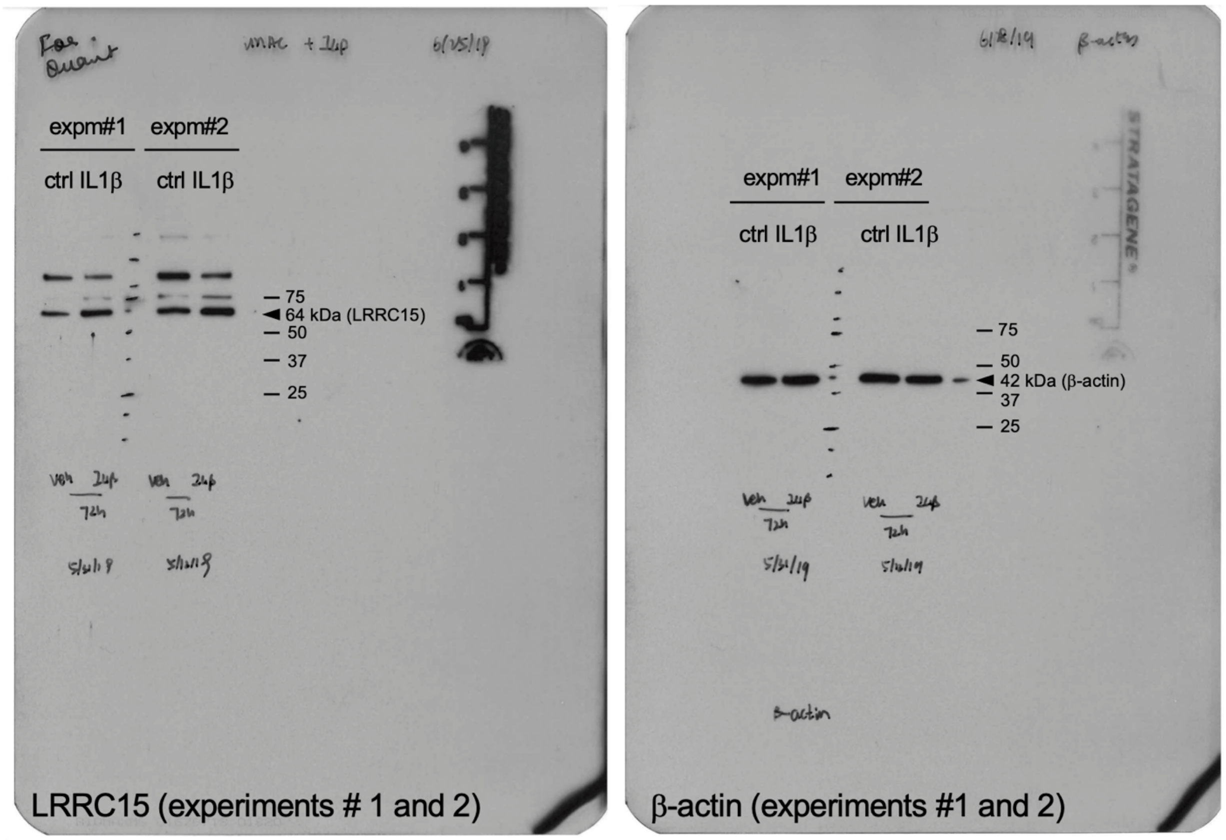

Supplementary Figure S9: Long-term cytokine treatment leads to sustained increased *Lrrc15* mRNA levels *in vitro*. (**A**) Experimental outline. Murine primary chondrocytes were left untreated or treated with 1ng/ml of IL1β for 2 weeks, with addition of IL1β with every medium change (every 72h). At 2 weeks, control- and IL1-treated cells were used for RNA isolation and RTqPCR analyses ("with IL1" group). A portion of the control- and IL1-treated cells were replated and kept in culture for an additional 2 weeks, with medium changes every 72h but without cytokine treatment. At 2 weeks, cells were used for RNA isolation and RTqPCR analyses ("after IL1" group). Created with BioRender.com. (**B**) RTqPCR analyses of control and IL1-treated cells (with IL1) and of the cells replated and kept in culture for 2 weeks (after IL1) showing that the long-term treatment with IL1 induces Lrrc15 mRNA expression, and that cells that were cultured in presence of IL1 for two weeks retained increased Lrrc15 mRNA levels relative to control cells, even after passage and after two weeks in culture without IL1 treatment and medium changes every 72h (n=4/ea). Data are shown as fold-changes vs. vehicle-treated controls (set as 1). ** indicates $p<0.01$ by *t*-test.

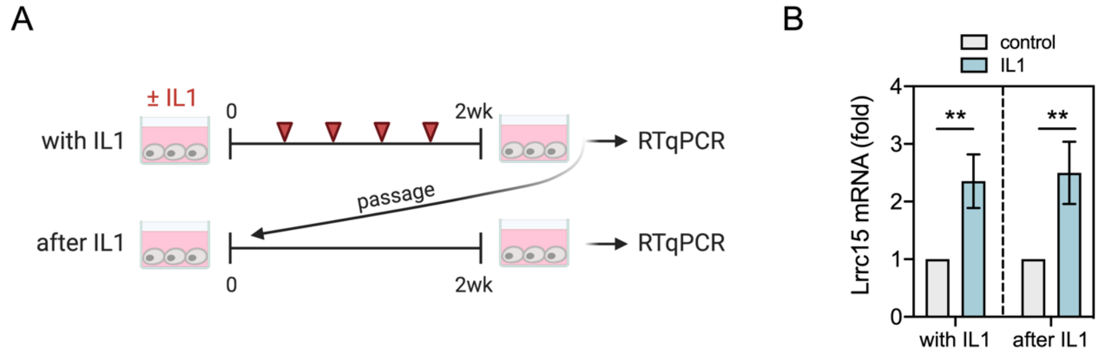

Supplementary Figure S10: Treatment with DNMT inhibitors leads to sustained (1 week) increased *Lrrc15* mRNA. RTqPCR analyses of (**A**) *Lrrc15* and (**B**) *Mmp13* mRNA expression in RNA isolated from monolayer cultures treated with vehicle (ctrl) or a combination of 2μM 5-Aza-2'-deoxycytidine and 40 nM trichostatin (5-aza). RNA was isolated at 1 week after treatment (n=3/each). Data are shown as fold-changes vs. vehicle-tread controls (set as 1). *indicates p<0.05 by *t*-test.

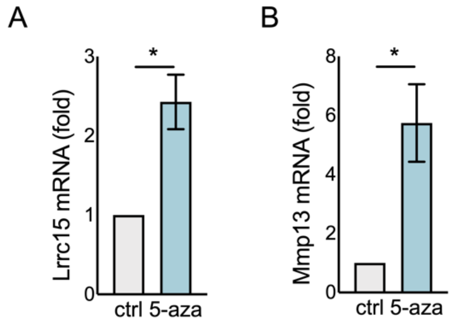

Supplementary Figure S11: Evaluation of the Lrrc15 knockdown in cells transfected with custom siRNA oligos. RTqPCR analyses of **(A)** *Lrrc15* (n=3/ea) and **(B)** *Lrrc17* (n=2/ea) mRNA expression in RNA isolated from monolayer cultures transfected with 25nM of a control non-targeting scrambled siRNA (siCtrl) or three different custom siRNAs targeting Lrrc15 (siLrrc15). RNA was isolated at 48 hours after transfection. Data are shown as fold-changes vs. mock transfected controls (set as 1). *indicates $p<0.05$, **indicates $p<0.01$ and ***indicates $p<0.001$ (vs. siCtrl) by ANOVA followed by Tukey's test. ns = not significant.

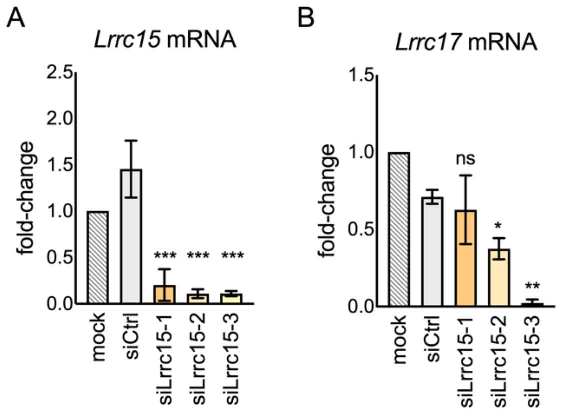

Supplementary Table S7: qPCR primers.

**Gene name**	**Forward 5'>3'**	**Reverse 5'>3'**	**Species**
Col2a1	CCAGCTGACCTCGCCACTGC	GGGTCCAGGCGCACCCTTTT	mouse
Eef1a1	GCCTTGGTTCAAGGGATGGA	ACAGTGCCAATGCCTCCAAT	mouse
Elf3	GGCCCTCATGGCTGCCACCT	TTGGGATCTTGTCTGAGGTCCTGGA	mouse
GAPDH	ATCAAGAAGGTGGTGAAGCA	GTCGCTGTTGAAGTCAGAGGA	human
Hprt1	TCCCAGCGTCGTGATTAGCGA	GGGCCACAATGTGATGGCCTCC	mouse
LRRC15	TGCTGATGTGTCCCTAGCAT	AGTGGCATAGCCTTGTCCAAA	human
Lrrc15	GAAGGCTCCCAATGAGTGCT	GATTCAGGGGCACAGGACAA	mouse
Lrrc17	AGATCCTGCTGCCTTTTTAGGG	CTGTAGTCACACCTCCAAGGG	mouse
Mmp10	GCAGCCCATGAACTTGGCCACT	AGGGACCGGCTCCATACAGGG	mouse
Mmp13	ATGGTCCAGGCGATGAAGACCCC	GTGCAGGCGCCAGAAGAATCTGT	mouse
Mmp3	TGTGTGCTCATCCTACCCATTGC	CCCTGTCATCTCCAACCCGAGGA	mouse
Nos2	TGCAACATGGGAGCCACAGCA	AGGGTGGTGCGGCTGGACTT	mouse
Ptgs2	CTGCTGCCCGACACCTTCAACA	CATTTCTTCCCCCAGCAACCCGG	mouse

Supplementary Table S8: Custom siRNA oligonucleotide sequences.

ID#		Sequence
**siLrrc15-1**	**sense**	5' C.A.G.A.A.U.A.C.U.U.C.A.U.A.A.G.G.A.A.U.U 3'
	**antisense**	5' U.U.C.C.U.U.A.U.G.A.A.G.U.A.U.U.C.U.G.U.U 3'
**siLrrc15-2**	**sense**	5' G.A.A.A.C.U.A.G.U.U.C.C.A.C.C.A.U.U.A.U.U 3'
	**antisense**	5' U.A.A.U.G.G.U.G.G.A.A.C.U.A.G.U.U.U.C.U.U 3'
**siLrrc15-3**	**sense**	5' C.C.C.U.G.A.A.G.A.U.G.G.A.G.A.A.G.A.A.U.U 3'
	**antisense**	5' U.U.C.U.U.C.U.C.C.A.U.C.U.U.C.A.G.G.G.U.U 3'
